# Genome-wide identification and expression analysis of the *GRAS* gene family under abiotic stresses in wheat (*Triticum aestivum* L.)

**DOI:** 10.1038/s41598-023-45051-0

**Published:** 2023-10-31

**Authors:** Shefali Mishra, Reeti Chaudhary, Bharti Pandey, Gyanendra Singh, Pradeep Sharma

**Affiliations:** 1 Deenbandhu Chhotu Ram University of Science and Technology, Murthal, India; 2 ICAR-Indian Institute of Wheat and Barley Research, Agrasain Marg, PO BOX-158, Karnal, Haryana India; 3 ICAR-National Dairy Research Institute, Karnal, India

**Keywords:** Plant biotechnology, Plant molecular biology

## Abstract

The GRAS transcription factors are multifunctional proteins involved in various biological processes, encompassing plant growth, metabolism, and responses to both abiotic and biotic stresses. Wheat is an important cereal crop cultivated worldwide. However, no systematic study of the *GRAS* gene family and their functions under heat, drought, and salt stress tolerance and molecular dynamics modeling in wheat has been reported. In the present study, we identified the *GRAS* gene in *Triticum aestivum* through systematically performing gene structure analysis, chromosomal location, conserved motif, phylogenetic relationship, and expression patterns. A total of 177 *GRAS* genes were identified within the wheat genome. Based on phylogenetic analysis, these genes were categorically placed into 14 distinct subfamilies. Detailed analysis of the genetic architecture revealed that the majority of *TaGRAS* genes had no intronic regions. The expansion of the wheat GRAS gene family was proven to be influenced by both segmental and tandem duplication events. The study of collinearity events between TaGRAS and analogous orthologs from other plant species provided valuable insights into the evolution of the *GRAS* gene family in wheat. It is noteworthy that the promoter regions of *TaGRAS* genes consistently displayed an array of *cis*-acting elements that are associated with stress responses and hormone regulation. Additionally, we discovered 14 miRNAs that target key genes involved in three stress-responsive pathways in our study. Moreover, an assessment of RNA-seq data and qRT-PCR results revealed a significant increase in the expression of *TaGRAS* genes during abiotic stress. These findings highlight the crucial role of *TaGRAS* genes in mediating responses to different environmental stresses. Our research delved into the molecular dynamics and structural aspects of GRAS domain-DNA interactions, marking the first instance of such information being generated. Overall, the current findings contribute to our understanding of the organization of the *GRAS* genes in the wheat genome. Furthermore, we identified *TaGRAS27* as a candidate gene for functional research, and to improve abiotic stress tolerance in the wheat by molecular breeding.

## Introduction

Wheat (*Triticum aestivum* L*.*) is an important cereal crop, contributing 20% of the daily caloric intake, is an rich source of protein and carbohydrates in the human diet. Grain yield is the most important economic parameter and is influenced by several biotic and abiotic factors. The demand for wheat is forecast to increase by 50% by 2050 (https://www.openaccessgovernment.org/demand-for-wheat/83189/). To cope with abiotic challenges such as salinity, drought, and heat, plants employ a variety of stress-responsive pathways and activated defense mechanisms^1^. Globally, these environmental constraints lead to average production losses of more than 50% in main crops^2^. To meet the food demands of the growing human population, breeding stress-tolerant varieties resistant to various environmental challenges, as well as improved quality and yield^3^ are future goals. Since traditional breeding has had little success in abiotic stress due to its multigenic nature and narrow genetic pool, using transgenic technology to develop stress-tolerant cultivars is a viable option. For the development of stress-tolerant plants, understanding the molecular mechanisms and exploring the stress-responsive genes that regulate plant responses to abiotic stresses^4, 5^. Transcription factors (TFs) are essential signaling elements in protecting plants from abiotic stress. Therefore, identifying relevant TFs is still essential for studying signaling pathways and abiotic stress. Translational genomics is a successful strategy to identify wheat genes.

After the first three functionally recognized members, gibberellic acid insensitive (GAI), a repressor of GAI (RGA), and a scarecrow (SCR), GRAS, a key TF gene family in plants, was verified^6–8^. The carboxyl terminus of GRAS proteins contains the highly conserved sequences LHR I, VHIID, LHR II, PFYRE, and SAW, which range in length from 400 to 770 amino acids^9–11^. These conserved motifs play an important role in protein function^12, 13^. For example, in Arabidopsis, mutations of the PFYRE and SAW motifs in the RGA and SLR1 regions cause major phenotypic alterations^8, 12^. GRAS proteins have more diverse amino-termini in length and sequence, implying functional specialisation^11^. By far, the *GRAS* gene family has been explored in several plant species^11^. Initially, based on shared features and sequence similarities, eight subfamilies of the GRAS gene family were identified: SCL3, SCR, SHR, LS, LISCL, PAT1, DELLA, and HAM. Each subfamily may have a unique function in a plant's physiological activities^11^. For example, the Arabidopsis SCR gene, which governs the radial form of the root, was the first GRAS member discovered^6^. Through the SCR/SHR complex, Arabidopsis SHR, a member of the GRAS gene family, is also necessary for root development^13–15^. AtSCL3 (SCARECROW-LIKE 3) has been demonstrated to positively control the gibberellin (GA) pathway and operate as a DELLA repressor, regulating GA homeostasis in root development. In comprehensive studies of their regulatory mechanisms, DELLA proteins were revealed to be repressors of GA signaling^8^. The members of the DELLA subfamily GAI, RGA, RGL1 (RGA-LIKE1), RGL2, and RGL3 can be distinguished from other GRAS proteins by the conserved DELLA motif in their N-terminal region. The PAT1 subfamily member linked to AtSCL13 is largely a positive regulator of phytochrome B (phyB) signaling, in contrast to other members of this subfamily, such as PAT1 and SCL21, which are involved in phytochrome A (phyA) signalling. Rice MONOCULM 1 (OsMOC1) expression in axillary buds is critical for regulating rice tillering^16^. Tomato (Ls) and Arabidopsis (LAS/SCL18) possess OsMOC1-like genes that are also important for the control of axillary meristem outgrowth^17^. BrLAS, a GRAS transcription factor from *Brassica rapa* L., is involved in drought stress tolerance in transgenic Arabidopsis^18^. Several GRAS members, including NSP1 and NSP2 (or their protein complex), are required for nodulation in *Medicago truncatula* Gaertn. as putative regulators of Nod-factor-inducible gene expression^19, 20^. Furthermore, NSP1 and NSP2 are necessary for strigolactone production in *M. truncatula* and *Oryza sativa* L.^5^. A recent study has shown that the GRAS transcription factors SCL6/SCL6-IV, SCL22/SCL6-III, and SCL27/SCL6-II are regulated by miRNA171 in Arabidopsis^21–23^. There are GRAS members with stress-related effects in *Arabidopsis*, cabbage (SCL13)^24^, rice (CIGR1 and CIGR2)^25^, tobacco (GRAS1)^26^, and poplar (SCL7)^27^, foxtail millet^28^, cucumber^29^. Researchers conducted a study to investigate the impact of abiotic stress on the DELLA gene, gibberellic acids (GA), and grain development in *Sorghum bicolor* (L.) Moench^30^. Similarly, studies on cucumber have shown that certain subfamilies such as SHR, SCR, and DELLA have a strong impact on the control of this species^29^. On the other hand , when studying cassava, scientists analysed how a specific gene called *MeGRAS* responds to various abiotic stresses, such as drought, salinity, cold stress, and exposure to hydrogen peroxide (H_2_O_2_)^31^. The findings of these studies strongly suggest that this gene plays diverse roles in cassava biology^31^.

Our study involved an extensive genome-wide study of 177 members of the wheat *GRAS* gene family. Identification of these GRAS genes was achieved through a multifaceted approach including their classification characterisation, expression profiling, and molecular dynamic simulations. To confirm the expression pattern obtained from *in-silico* analysis, we performed a quantitative real-time polymerase chain reaction. The findings strongly suggest that *TaGRAS27* is a promising candidate for enhancing drought, heat, and salt tolerance through genome-editing techniques. The results of this study are important for the systematic elucidating of the functional roles played by the TaGRAS family genes in wheat.

## Materials and methods

### Identification and annotation of *TaGRAS *genes in *T. aestivum*

To identify the GRAS genes in wheat, the Phytozome database^32^ and the Rice Annotation Project (RAP) (https://rapdb.dna.afrc.go.jp/) was used to identify the GRAS protein sequences of related plant species, viz *Arabidopsis thaliana* (L.) Heynh.*, Brassica napus* L.*,* and *Hordium vulgare* L. The bread wheat proteome sequences (fp://fp.ensemblgenomes.org/pub/-plants/release-51/fasta/triticumaestivum/pep/) were used as the database for BLASTp^33^, while sequences from other species, such as *A. thaliana*, were used as query sequences *O. sativa, B. napus* and *H. vulgare* are used. These techniques were used to identify potential TaGRAS candidates. After eliminating duplicate results, the remaining sequences were scanned using HMMscan (https://www.ebi.ac.uk/Tools/hmmer/search/hmmscan), the SMART database^34^ (http://smart.embl-heidelberg. de/), NCBI CDD^35^ and pfam^36^ among others to confirm the GRAS domain (PF03514) (http://pfam.Sanger.Ac.United Kingdom/). Calculations of the number of amino acids, molecular weights (MW), and isoelectric points (pI) of each TaGRAS protein were also performed using ExPASy tools (http://www.expasy.ch/tools/pitool.html).

### Phylogenetic analysis and classification of the *TaGRAS* gene family

Clustal W software was used to analyse all GRAS protein sequences from rice, Arabidopsis, brassica, and wheat^37^. Following that, an unrooted phylogenetic tree was built in MEGA-X with 1000 bootstrap repetitions using Maximum likelihood (ML) using default parameters e.g., used (JTT model) with uniform rates and 4 number of threads^38^. TaGRAS members in wheat were classified into subfamilies based on their Arabidopsis and rice homologues.

Using Blast2GO mapping (https://www.blast2go.com/) for molecular functions, biological processes and cellular components, it was possible to identify the functional annotation of the target genes. Metabolic pathways were annotated using maps from the KEGG database^39^.

### Motif prediction and Gene structure

The MEME program was used for the conserved motif analysis of GRAS^40^, whose parameters were set as the standard. Annotation and visualization of identified motifs were performed using the TBtools^41^. The online tools Gene Structure Display Server (GSDS 2.0; http://gsds.gao-lab.org/)^42^ based on the CDS and matching to genetic sequences were also used to study the exon–intron patterns.

### Chromosomal locations, Orthologous events, and interaction network analysis

Using the *T. aestivum* genome annotation dataset, the chromosomal locations of all TaGRAS members were verified^43^. To find the orthologous genes, wheat *TaGRAS* gene sequences were blasted by BLASTn^33^ using parameters e-value of 1e−10 to detect duplication of genes. Using MCScanX's default settings, the pattern of duplicated TaGRAS was categorized as segmental and tandem duplications^44^. A region of the chromosome that is smaller than 200 kb and has two or more genes is known as a tandem duplication^45^. However, synonymous (K_s_) and non-synonymous (K_a_) substitutions of each duplicated *TaGRAS* gene as well as synteny between interspecies were determined by TBtools^41^. The STRING v1054 databases (https://string-db.org/) were used to identify functional protein–protein interactions^46^. The GRAS protein sequences were input into the STRING^46^ program, and *H. vulgare* was used as the reference species to search in the database. With blast software set to an e-value of 1e−10, the *H. vulgare* genome was searched against all known interaction partners. The best-hit gene for each gene was chosen using Cytoscape58 to create a PPI network^47^. Finally, Cytoscape cyto-Hubba plugin was used to determine the top hub genes from the interaction network (Cytoscape Consortium 2016).

### TaGRAS promoter analysis

*Cis*-elements are important in controlling plant growth and environmental adaptation. The sequences 1500 bp upstream of the start codon were extracted, used as a hypothetical promoter and then submitted to the online tool PlantCARE (http://bioinformatics.psb.ugent.be/webtools/plantcare/html/) to predict the *cis*-elements and identify the putative *cis*-elements in the promoter regions of TaGRAS^48^.

### Prediction of *miR* genes targeting TaGRAS

The Ensembl plant database was used to find the transcript sequences of *TaGRAS* gene. Now the transcript sequences of TaGRAS and the matured miRNA sequences^49^ were examined using the default parameters of the psRNATarget service^50, 51^.

### RNA-seq derived gene expression profiling

To identify the in-silico gene expression patterns across a range of tissues under control and stressed conditions, RNAseq data was utilised. We acquire transcripts per million (TPM) values for each *TaGRAS* from the expVIP database (http://www.wheat-expression.com/), and created heatmaps to visually represent these expression profiles using Clustvis 2.0^52^ (https://biit.cs.ut.ee/clustvis/). Furthermore, we validate the expression of 20 *GRAS* genes identified by *in-silico* analysis under abiotic stress conditions using qRT-PCR as described in section "RNA isolation and real-time quantitative PCR analysis.".

### Molecular modelling and molecular dynamics (MD) simulations

The three-dimensional structure was generated using the Rosettafold^53^ as it employs deep learning techniques for accurate prediction of protein structure without relying on homology. MD simulations were carried out for the modeled GRAS protein structures using the Desmond MD simulation package (release 2018) of Schrodinger (Desmond Research) to investigate the conformational changes of the protein with the solvent system. The OPLS_2005 force filed^54^ was used for the proteins. Utilizing the system builder module of Desmond, the proteins were solvated in the cubical water box (TIP3P water model) keeping a 10 Å distance between the box edge and protein atom. Systems were neutralized by the addition of counter ions and 0.15 M ionic concentration was maintained by the addition of Na^+^ and Cl ions. The minimization of the solvated built systems was performed with 10,000 steepest descent steps followed by gradual heating from 0 to 300 K, under Berendsen NVT ensemble^55^. Prior to the production run, the systems underwent heat relaxation for 5 ns using the Nose–Hoover Chain thermostat^56^ and Martyna-Tobias-Klein barostat method for maintaining temperature and pressure scale at 300 K and 1 atm. Lastly, a 20 ns production run was carried out for each system using a cut-off distance of 12 Å for non-bonded interactions. The coordinates of the simulations were recorded every 10 ps. The figures were rendered through UCSF chimera^57^.

### Plant material and growth conditions

In the current study, seeds of six contrasting wheat genotypes (C306, WL711, RAJ3765, HS240, HD2009, and KRL213) were obtained from the Germplasm Unit of the ICAR-Indian Institute of Wheat and Barley Research in Karnal, India. Under controlled conditions, the seeds were germinated in Petri dishes at 22 °C after being sterilized with 1% sodium hypochlorite for 10 min and washed three times with distilled water. After five days of germination, seedlings were transferred to full-strength Hoagland's solution phyta-jars and incubated for 14 days in a BOD incubator with two sets of three biological replicates of each genotype. To study the effects of drought stress, two different wheat cultivars C306 and WL711-which are drought tolerant and susceptible, respectively, were used. At the seedlingstage, an osmotic stress using 20% (v/v) polyethylene glycol (PEG) 6000 was given after growing in Hoagland's solution for 14 days, and samples were collected at time intervals of 0 h, 3 h, 24 h, and 48 h. Unstressed seedlings served as the control^58^. For the expression study, leaf samples from control and stressed seedlings were also collected at the aforementioned intervals. Two contrast wheat genotypes RAJ3765 (heat tolerant) and HS240 (heat sensitive) were selected for heat stress. These plants were kept at a basal temperature of 37 °C for 3 h, and then kept at room temperature for 3 h, and finally 42 °C for 3 h (at Acquired). Leaf samples from the baseline and acquired stress levels were taken at the time interval mentioned above. For salt stress, two contrasting wheat genotypes HD2009 (salt sensitive) and KRL213 (salt tolerant) were used. Both genotypes at two leaves seedling stage were stressed with 150 mM NaCl. After the treatment, the leaf samples were taken at 0, 3, 24, and 48 h. For total RNA isolation, all acquired samples were immediately wrapped in foil and frozen in liquid nitrogen at − 80 °C.

### RNA isolation and real-time quantitative PCR analysis

TRIzol reagent (Thermo Scientific, USA) was used to isolate the total RNA as per manufacturer's instructions. The extracted RNA was subjected to DNase I (NEB, USA) treatment. RNA integrity was checked by 1.2% native agarose gel electrophoresis and concentration was measured using the Nano-Drop ND-1000 spectrophotometer (Thermo Scientific, USA). Using Superscript-III reverse transcriptase (Thermo Scientific, USA), 1 µg of total RNA was converted into the first strand of cDNA. PCR reactions were carried out on BIO-RAD CFX96 Touch Real-Time PCR System (Bio-Rad, USA) in 10 µl reaction volumes, comprising 5 µl of 2 × SYBR Green Master Mix (Thermo Scientific, USA), 0.5 µl of each primer (0.5 µM) and 1 µl of the cDNA diluted to a ratio of 1:2. The primers were designed using the PrimerBlast tool^59^, with a final product size of approximately 150 base pairs (Supplementary Table S1). The primer efficiency was calculated by using the formula: E = 10 ^(-1/slope)^^60^. The thermal cycling conditions for qRT-PCR was: 95 °C for 5 min, then 40 cycles of 94 °C for 15 s, 55 °C for 30 s and 72 °C for 30 s, and a final melting curve step from 65 °C to 95 °C with a ramp speed of 0.5 °C per 5 s. Actin, a housekeeping gene, was used as an internal control^61^ (F: 5′-GGAGAAGCTCGCTTACGTG-3′ & R: 5′-GGGCACCTGAACCTTTCTGAA-3′). The standard deviation was measured by averaging the threshold cycle values (Ct) of the experimental triplicates. The comparative 2^∆∆−Ct^ method was used to determine the relative expression level of genes^62^.

### Ethical approval

The use of plant parts in the study complies with institutional guidelines.

## Results

### Identification and annotation of *TaGRAS* genes in wheat

In total, we identified 177 wheat genes (Supplementary Table S2) belonging to the *GRAS* gene family, which were designated as *TaGRAS1* to *TaGRAS180*. However, it should be noted that the sequence lengths of three genes, namely *TaGRAS71, TaGRAS72*, and *TaGRAS103*, were relatively short, posing challenges to the alignment procedure. Therefore, to facilitate the study, these genes were excluded from further analysis. As a result, our current investigation focused on the remaining 177 GRAS genes. For the comparative genomic evaluation, GRAS protein sequences were retrieved from the reference genomes of four different species (Supplementary Table S3) and 422 GRAS proteins were identified. These proteins were used to assess the expression of GRAS transcription factors that reflect the major evolutionary divergence of wheat species. The protein molecular weight (MW) of the identified GRAS family members ranged from 16.76596 kDa to 163.7962 kDa, and the isoelectric point (pI) ranged from 4.8 to 9.0. However, the theoretical pI values of 23 proteins were higher than 7, and most proteins contained acidic amino acids (Supplementary Table S4).

### Analysis of phylogenetic tree of GRAS transcription factor

**Figure 1:**
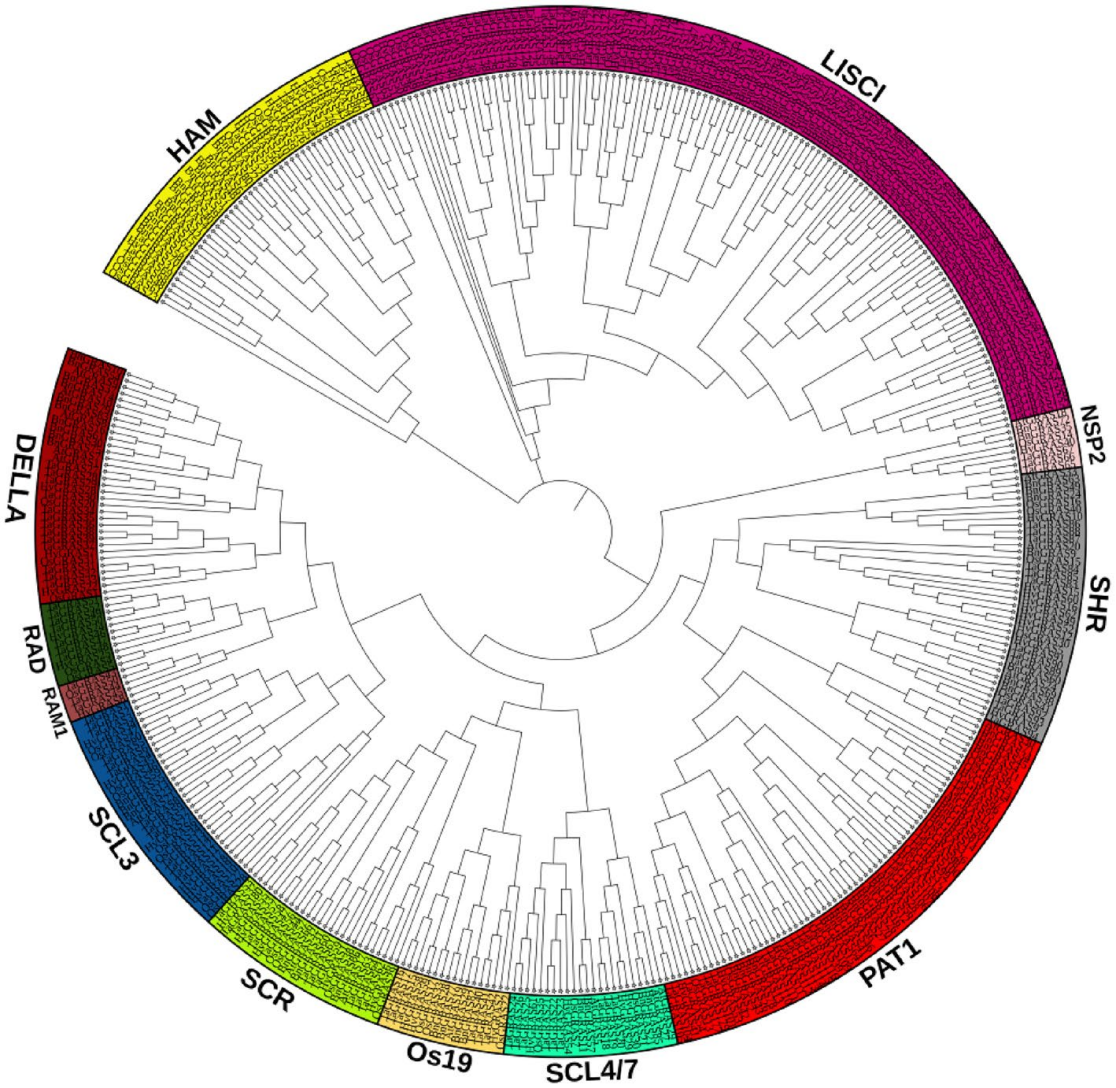
Characterised sequences of five species (wheat, Arabidopsis, rice, barley and oilseed rape) were used to draw this phylogenetic tree (Maximum Likelihood) with the wheat GRAS proteins using MEGA X^34^. Combined phylogenetic analysis of GRAS proteins from *T. aestivum* (Ta), *A. thaliana* (At), *O. sativa* (Os), *H. vulgare* (Hv) and *B. napus* (Bn). The GRAS proteins are clustered into 14 subgroups, marked by different colours.

The phylogenetic tree was constructed based on the maximum likelihood method to analyse the evolutionary correlation of *GRAS* genes (Fig. 1). *TaGRAS* genes were classified into fourteen groups. Group-I (LISCL subfamily) comprised 61 members from the wheat, whereas 9 proteins were from Arabidopsis and 19, 15, and 19 were from rice, barley and oilseed rape, respectively. Group-II (HAM subfamily) consisted of 13, 7, 4, 6 and 13 GRAS proteins in wheat, rice, Arabidopsis, barley, and oilseed rape, respectively. Like the Arabidopsis GRAS proteins have distinct and necessary biological functions in the HAM subfamily that are required for maintaining the shoot apical meristem^63^. Whereas, 12 TaGRAS proteins from *T. aestivum*, belong to Group-III (SHR subfamily). However, in Group-IV, PAT1, consists of 23, 5, 5, 5, and 17 individuals from the different crops of wheat, rice, *A. thaliana*, barley, and oilseed rape, respectively. Like *A. thaliana*, three GRAS proteins (TaGRAS106, TaGRAS107, and TaGRAS108) may also play significant roles in the wheat phytochrome signal transduction pathways. Group-V includes three proteins TaGRAS65, TaGRAS66, and TaGRAS67, Group-VI of TaGRAS40, TaGRAS41 and TaGRAS42 and Group-VII of TaGRAS15, TaGRAS16, and TaGRAS17of wheat proteins (Fig. 1). Group-VIII includes the SCR protein, having two GRAS proteins from Arabidopsis (AthGRAS21 and AtGRAS29) and thirteen GRAS proteins from wheat. Group-IX and -X (RAM 1 and RAD subfamily) are basically involved in mycorrhizal signalling. Three wheat genes (*TaGRAS43*, *TaGRAS44* and *TaGRAS45*) and one each in rice (*OsGRAS50*) and barley gene (*HvGRAS8*) were clustered in RAM1 subfamily. However, seven genes from wheat, two each from rice and barley were grouped in the RAD subfamily. Thirteen wheat proteins comprised of all DELLA proteins formed Group-XI. According to prior reports on Arabidopsis, the majority of these proteins including RGL2 (AtGRAS9), RGL3 (AtGRAS10), GAI (AtGRAS3), and RGA, are negative regulators of GA signalling (AtGRAS7)^8, 64–67^. The rest of the Groups-XII, XIII and XIV had 3, 6 and 14 genes, respectively.

To assess the activities of the *TaGRAS* genes, we examined the enrichment of GO (Gene Ontology) and KEGG (Kyoto Encyclopaedia of Genes and Genomes) terms. The 177 TaGRAS family members were classified into three ontology categories based on GO enrichment analysis: biological process, cellular component, and molecular function (Supplementary Table S5). From these GO terms, 30 functional terms were then created (Supplementary Fig. 1). With 146 genes involved in biological regulation, cellular activities, and metabolic processes, we expected that the *TaGRAS* gene family in wheat is involved in many plant regulatory processes. In addition, 26 genes revealed putative transcription factor activity and binding roles.

### Analysis of gene structures and conserved motifs

To investigate the properties of the *GRAS* genes, all of the *TaGRAS* genes were utilized to assess the distribution of exons, introns, and UTR (Supplementary Fig. 2). The analysis revealed that most *TaGRAS* genes were intron-less 72.8% (129 out of 177) particularly, all genes in Os19, DLT, HAM, NSP2, SCL4/7, and RAD were intron-less. However, DELLA and SHR subfamilies were intron-less except two genes each (*TaGRAS30*, *TaGRAS32*, and *TaGRAS85*, *TaGRAS87*, respectively). The LAS subfamily also has no intron genes except one gene (*TaGRAS21*), whereas, members of LISCL, PAT1, SCR, and RAM1 subfamilies showed numerous introns (Supplementary Fig. 2). Additionally, the intron of the SCL3 subfamily member TaGRAS3 was exceptionally lengthy, covering upto 1000 bp. In general, gene structure varied across members of the same subfamily, indicating gene family diversification. The conserved motifs for each GRAS protein family were discovered (Supplementary Fig. 3) and concluded that the majority of GRAS proteins in the same group had similar motifs. MEME was used to determine the LOGO of these protein motifs (Supplementary Table S6; Supplementary Fig. 4). The GRAS domain, a common structure, was found in the TaGRAS. To understand each group, fourteen motifs were identified and utilised. Members of the same group were highly similar as far as motif composition but differed from other groups. The C-terminal portion of the GRAS proteins appeared to be more conserved than the N-terminal, as evidenced by the likelihood motifs^9^.

### Chromosome distribution of the GRAS transcription factor family

**Figure 2:**
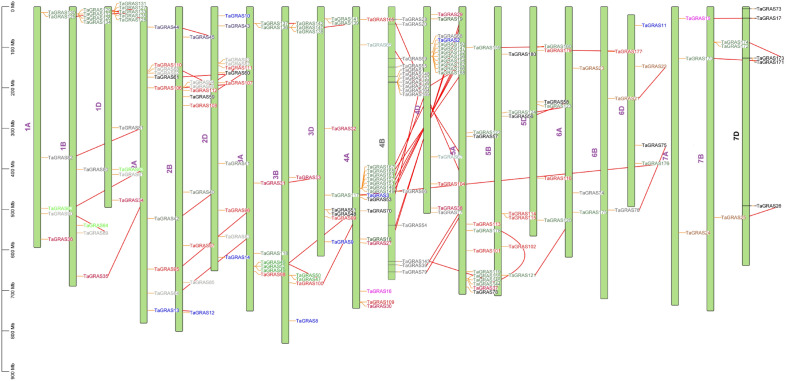
Distribution of *TaGRAS* genes on 21 wheat (*T. aestivum*) chromosomes constructed using Mapchart^38^.

The physical location map of the *TaGRAS* was drawn based on the physical location information of the wheat genome. 177 *TaGRAS* genes were widely and irregularly distributed on the 21 chromosomes of *T. aestivum* (Fig. 2). Chromosomes 4A (n = 17, 9.6%), 4B (n = 16, 8.9%), and 4D (n = 16, 8.9%) each contained the majority of the GRAS transcription factors. By contrast, chromosome 2A, 2B contains 10 genes (5.6%) and 2D has 11 genes (6.11%). whereas many GRAS transcription factors were gathered at chromosomes 5A, 5B and 5D (13, 8, 7) respectively. However, 9, 8, and 8 GRAS transcription factors were also located on chromosomes 3A, 3B, and 3D, respectively. Chromosome 1A has 6 genes, chromosome 1B contains 8 genes and chromosome 1D has 9 genes (Fig. 2). The localization of the GRAS transcription factor was lower on chromosome 6 and 7.

### microRNA targeting GRAS transcription factors in wheat

Previously, it was found that three Arabidopsis *GRAS* genes were targeted by miRNA171^68^. We identified 10 *TaGRAS* genes targeted by miRNA171 in wheat (*TaGRAS82*, *TaGRAS81*, *TaGRAS80*, *TaGRAS79*, *TaGRAS77*, *TaGRAS78*, *TaGRAS74*, *TaGRAS76*, *TaGRAS75*, and *TaGRAS73*), which were located on various chromosomes e.g., 1A, 1B, 1D, 4A, 4B, 4D, 5A, 6A, 6B, 6D. 7A, and 7D. In our analysis, miRNA171 was complementary to the internal region of these twelve GRAS transcription factors. These proteins belong to the HAM subfamily, which is necessary for maintaining the shoot apical meristem. miRNA160 targets PAT1 subfamily (*TaGRAS95*, *TaGRAS96* and *TaGRAS97*), basically participate in phytochrome signaling. miRNA164 targets four genes that participating in nodulation signalling pathways (Table1). However, miR399 targeted different GRAS subfamilies i.e., SHR, LISCL, DLT and DELLA. Whereas, miR167 and miR397-5p target the SCR subfamily and is involved in radial patterning in both embryonic roots and shoots.

**Table 1:** Prediction of Tae-miR genes and their targets by using the psRNATarget server with default parameters.

miRNA_Acc	Gene Ids	Target_start	Target_end	miRNA_aligned_fragment	Target_aligned_fragment	Inhibition
tae-miR171a	TaGRAS77	1157	1177	UGAUUGAGCCGUGCCAAUAUC	GAUAUUGGCACGGCUCAAUCA	Cleavage
	TaGRAS79	1151	1171	UGAUUGAGCCGUGCCAAUAUC	GAUAUUGGCACGGCUCAAUCA	Cleavage
	TaGRAS78	888	908	UGAUUGAGCCGUGCCAAUAUC	GAUAUUGGCACGGCUCAAUCA	Cleavage
	TaGRAS76	1083	1103	UGAUUGAGCCGUGCCAAUAUC	GAUAUUGGCGCGGCUCAAUCA	Cleavage
	TaGRAS73	564	584	UGAUUGAGCCGUGCCAAUAUC	GAUAUUGGCGCGGCUCAAUCA	Cleavage
	TaGRAS75	1426	1446	UGAUUGAGCCGUGCCAAUAUC	GAUAUUGGCGCGGCUCAAUCA	Cleavage
	TaGRAS74	1433	1453	UGAUUGAGCCGUGCCAAUAUC	GAUAUUGGCGCGGCUCAAUCA	Cleavage
	TaGRAS82	1268	1288	UGAUUGAGCCGUGCCAAUAUC	GAUAUUGGCGCGGCUCAAUUA	Cleavage
	TaGRAS81	1124	1144	UGAUUGAGCCGUGCCAAUAUC	GAUAUUGGCGCGGCUCAAUUA	Cleavage
	TaGRAS80	1131	1151	UGAUUGAGCCGUGCCAAUAUC	GAUAUUGGCGCGGCUCAAUUA	Cleavage
tae-miR160	TaGRAS96	166	186	UGCCUGGCUCCCUGUAUGCCA	UCGCCUGCAUGGAGCCCGGCG	Cleavage
	TaGRAS95	163	183	UGCCUGGCUCCCUGUAUGCCA	CUGCCGGCAUGGAGCCGGGCG	Cleavage
	TaGRAS97	177	197	UGCCUGGCUCCCUGUAUGCCA	UCGCCUGCAUGGAGCCGGGCG	Cleavage
	TaGRAS149	685	705	UGCCUGGCUCCCUGUAUGCCA	AAGGAGAGGGGGGGCCAGGCG	Cleavage
	TaGRAS65	347	367	UGGAGAAGCAGGGCACGUGCA	CGGACGGCCCCUGCUACUCCA	Cleavage
	TaGRAS67	348	368	UGGAGAAGCAGGGCACGUGCA	CGGACGGUCCCUGCUACUCCA	Cleavage
	TaGRAS66	327	347	UGGAGAAGCAGGGCACGUGCA	CGGACGGUCCCUGCUACUCCA	Cleavage
tae-miR395b	TaGRAS116	1905	1924	UGAAGUGUUUGGGGGAACUC	UAGGUACCACAAGCAUUUCA	Cleavage
	TaGRAS145	1692	1711	UGAAGUGUUUGGGGGAACUC	UAGGUACCACAAGCAUUUCA	Cleavage
tae-miR399	TaGRAS102	4202	4220	UGCCAAAGGAGAAUUGCCC	UGGCACUUCUUUUCUGGCG	Cleavage
	TaGRAS49	1204	1222	UGCCAAAGGAGAAUUGCCC	GAGCAGUUCUACUAUGGCG	Cleavage
	TaGRAS38	38	56	UGCCAAAGGAGAAUUGCCC	GGCCAUUUCUCCUUCGGCU	Cleavage
tae-miR408	TaGRAS85	1429	1449	CUGCACUGCCUCUUCCCUGGC	UUCGGGGGCGAGGCGGUGGAG	Cleavage
	TaGRAS84	1390	1410	CUGCACUGCCUCUUCCCUGGC	UUCGGGGGCGAGGCGGUGGAG	Cleavage
	TaGRAS17	315	335	CUGCACUGCCUCUUCCCUGGC	GCCGGCGAGGAGGCUCUGCAG	Cleavage
	TaGRAS83	1496	1516	CUGCACUGCCUCUUCCCUGGC	UUUGGGGGCGAGGCGGUGGAG	Cleavage
	TaGRAS27	712	732	CUGCACUGCCUCUUCCCUGGC	GCCUGCGCGGAGGCCGUGCAG	Cleavage
	TaGRAS143	1292	1312	CUGCACUGCCUCUUCCCUGGC	CUAAUGGAGGAGGCAGGGCAC	Cleavage
	TaGRAS144	1267	1287	CUGCACUGCCUCUUCCCUGGC	CUAAUGGAGGAGGCAGGGCAC	Cleavage
	TaGRAS90	1527	1546	CUGCACUGCCUCUUCCCUGGC	CACGUGGAAGGGGCA-UGCAG	Cleavage
	TaGRAS91	1411	1430	CUGCACUGCCUCUUCCCUGGC	CACGUGGAAGGGGCA-UGCAG	Cleavage
	TaGRAS89	1389	1408	CUGCACUGCCUCUUCCCUGGC	CACGUGGAAGGGGCA-UGCAG	Cleavage
tae-miR530	TaGRAS114	3190	3210	UGCAGUGGCAUAUGCAACUCU	AGGGGUGCAUGUGCUGAUGCA	Cleavage
	TaGRAS113	3270	3290	UGCAGUGGCAUAUGCAACUCU	GGGGGUGCAUGUGCUGAUGCA	Cleavage
tae-miR1119	TaGRAS92	852	875	UGGCACGGCGUGAUGCUGAGUCAG	CUUCGUCGACCUCACGCCGUGGCA	Cleavage
	TaGRAS94	591	614	UGGCACGGCGUGAUGCUGAGUCAG	CUUCGUCGACCUCACGCCGUGGCA	Cleavage
	TaGRAS121	625	647	UGGCACGGCGUGAUGCUGAGUCAG	CUGAUUGGCAGCACGCCGAGCCC	Cleavage
	TaGRAS119	300	322	UGGCACGGCGUGAUGCUGAGUCAG	CUGAUUGGCAGCACGCCGAGCCC	Cleavage
	TaGRAS120	621	644	UGGCACGGCGUGAUGCUGAGUCAG	GCGGAUUGGCAGCACGCCGAGCCC	Cleavage
tae-miR159a	TaGRAS178	375	395	UUUGGAUUGAAGGGAGCUCUG	AAGUGUCUUCUUCGAUCCAAC	Cleavage
tae-miR167a	TaGRAS64	1248	1268	UGAAGCUGCCAGCAUGAUCUA	GGGAACGUGCUGGCGGUUGCC	Cleavage
	TaGRAS62	1247	1267	UGAAGCUGCCAGCAUGAUCUA	GGGAACGUGCUGGCGGUUGCC	Cleavage
tae-miR319	TaGRAS132	1939	1959	UUGGACUGAAGGGAGCUCCCU	CAGCAGCUUCCUGUGGUUCAG	Cleavage
tae-miR397-5p	TaGRAS180	2011	2031	UCACCGGCGCUGCACACAAUG	GGUUCCGUGCGGCGUCGCUGG	Cleavage
	TaGRAS55	2012	2032	UCACCGGCGCUGCACACAAUG	GGUUCCGUGCGGCGUCGCUGG	Cleavage
	TaGRAS53	1857	1877	UCACCGGCGCUGCACACAAUG	GGUUCCGUGCGGCGUCGCUGG	Cleavage
tae-miR398	TaGRAS5	986	1006	UGUGUUCUCAGGUCGCCCCCG	GAGGCGCGGCUCGAGGACAUG	Translation
tae-miR444a	TaGRAS155	1030	1050	UUGCUGCCUCAAGCUUGCUGC	CGGGCAGGCCUGAGGCAGCUG	Cleavage
	TaGRAS44	980	1000	UUGCUGCCUCAAGCUUGCUGC	ACAGCAUGCUGGGGUCAGUAA	Translation
	TaGRAS43	980	1000	UUGCUGCCUCAAGCUUGCUGC	ACAGCAUGCUGGGGUCAGUAA	Translation
	TaGRAS154	1639	1659	UUGCUGCCUCAAGCUUGCUGC	CGAGCAGGCCUGAGGCAGCUG	Cleavage
	TaGRAS109	1030	1050	UUGCUGCCUCAAGCUUGCUGC	CGAACAAGUUUGGGGCAUCAA	Cleavage

### Identification of orthologous and paralogous *GRAS* genes

**Figure 3:**
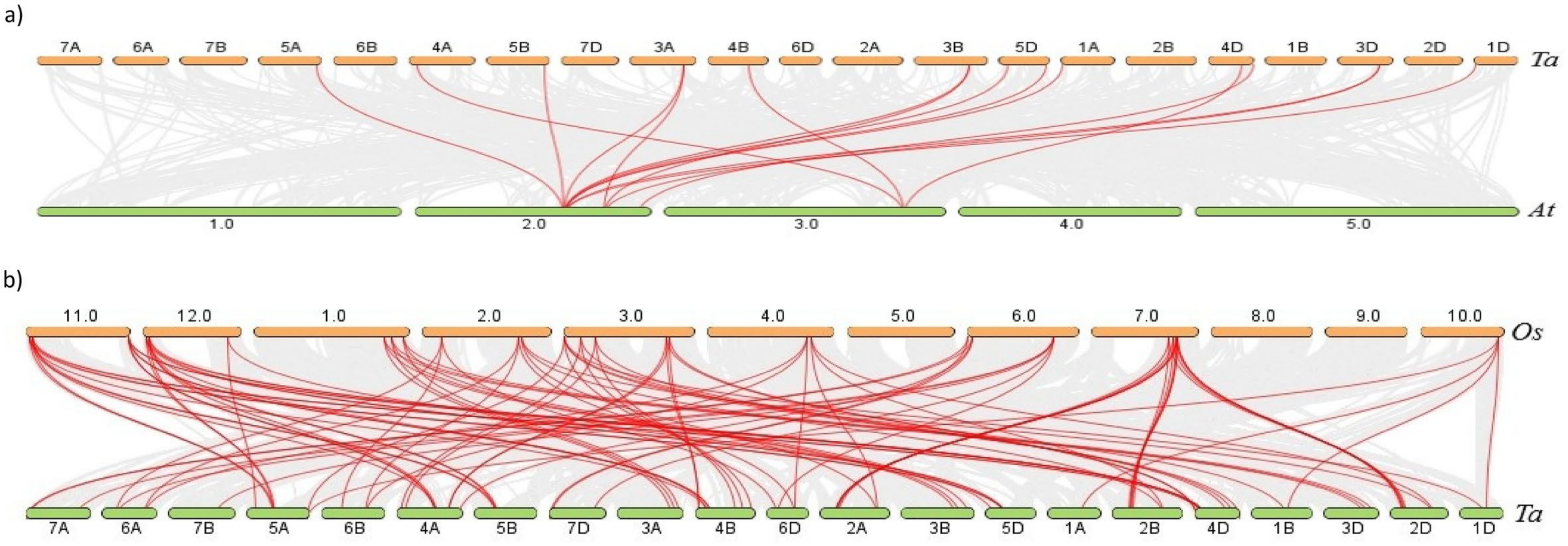
(**a**) Interspecies synteny of *T. aestivum* and *A. thaliana* (**b**) *T. aestivum* and *O. sativa* on the basis of orthologous genes. Gray lines in the background indicate the collinear blocks within *T. estivum* (Ta) and *A. thaliana* (At), *T. aestivum* (Ta) and *O. sativa* (Os) while the red lines highlight the syntenic GRAS gene pairs by using TBtools^41^.

Comparative analysis was utilized to evaluate the orthologous *GRAS* gene triplication in wheat and *Aegilops tauschii*. We discovered 85 orthologous gene pairs among all the GRAS proteins from wheat and *A. tauschii*. Contrarily, there were 124 GRAS orthologous gene pairs discovered between wheat and rice, 117 between wheat and *Triticum dicoccoides*, and just 8 between wheat and Arabidopsis (Supplementary Table S7). We discovered that each rice *GRAS* gene contained several wheat orthologs among the orthologous gene pairs, indicating that many GRAS transcription factors in wheat experienced duplication along with genome triplication. Since wheat and rice have a close lineage, there are more GRAS orthologous genes between the two than between wheat and Arabidopsis (Fig. 3). Additionally, in wheat, Arabidopsis, and rice, only 60, 7, and 22 paralogous *GRAS* gene pairs, respectively were found. Analysis of collinearity relationships can provide orthologous relationships between different species. GRAS gene pairings between the genomes of *T. aestivum* and *A. thaliana* were synthesized. The findings revealed that 16 *T. aestivum GRAS* genes shared syntenic relationships with *AtGRAS* genes (Fig. 3A), indicating that these genes may have aided in the development of the *TaGRAS* gene family. Similarly, Fig. 3B showed syntenic relationships with *OsGRAS* (Fig. 3B).

However, K_s_ values, K_a_ values, K_a__K_s_ ratios, and divergence times of paralogous and orthologous GRAS family genes were calculated to assess the effectiveness of evolutionary constraints (Table 2). K_a__K_s_ ratios were generally greater than 1 for segmentally duplicated *TaGRAS* gene pairs. Moreover, segmental gene divergence occurred between 18.76 and 34.97 Mya. These results suggested that the *TaGRAS* gene family may have been the target of strong purifying selection during evolution.

**Table 2:** Ka_Ks of GRAS gene on the basis of gene pairs.

Seq_1	Seq_2	Ka	Ks	Ka_Ks	Time (MYA)
TaGRAS2	TaGRAS3	0.045236	0.151092	0.299395	11.51612
TaGRAS5	TaGRAS6	0.005693	0.065313	0.087159	4.978141
TaGRAS7	TaGRAS9	0.012698	0.137515	0.092341	10.48134
TaGRAS12	TaGRAS13	0.043409	0.086674	0.500829	6.606255
TaGRAS15	TaGRAS17	0.01797	0.08487	0.211742	6.468716
TaGRAS19	TaGRAS18	0.012927	0.087091	0.14843	6.638018
TaGRAS22	TaGRAS21	0.014475	0.060128	0.240729	4.582953
TaGRAS26	TaGRAS25	0.005453	0.032232	0.169169	2.456671
TaGRAS27	TaGRAS29	0.005274	0.09583	0.055031	7.304141
TaGRAS31	TaGRAS33	0.01352	0.123547	0.109432	9.416666
TaGRAS34	TaGRAS35	0.014325	0.032408	0.442002	2.470143
TaGRAS38	TaGRAS39	0.031208	0.06991	0.446407	5.328531
TaGRAS40	TaGRAS42	0.023753	0.10042	0.236537	7.65397
TaGRAS44	TaGRAS45	0.005024	0.109193	0.046009	8.32266
TaGRAS46	TaGRAS47	0.004328	0.083475	0.051849	6.362457
TaGRAS51	TaGRAS52	0.006975	0.106518	0.065484	8.118762
TaGRAS56	TaGRAS58	0.003233	0.116024	0.027863	8.843295
TaGRAS60	TaGRAS61	0.005097	0.052245	0.097556	3.982126
TaGRAS62	TaGRAS63	0.02355	0.135294	0.174064	10.31204
TaGRAS65	TaGRAS67	0.004815	0.092117	0.052268	7.021135
TaGRAS69	TaGRAS70	0.019612	0.113916	0.172161	8.682652
TaGRAS75	TaGRAS76	0.006484	0.061122	0.10609	4.658684
TaGRAS77	TaGRAS79	0.007216	0.078077	0.092421	5.950986
TaGRAS81	TaGRAS82	0.004368	0.081428	0.053647	6.206431
TaGRAS83	TaGRAS84	0.014326	0.066018	0.216994	5.031897
TaGRAS86	TaGRAS88	0.001531	0.065529	0.023361	4.994592
TaGRAS89	TaGRAS90	0.00992	0.070773	0.14017	5.394289
TaGRAS93	TaGRAS94	0.001953	0.076053	0.025681	5.796718
TaGRAS95	TaGRAS96	0.013962	0.071059	0.196491	5.416084
TaGRAS99	TaGRAS100	0.00475	0.121678	0.039037	9.274234
TaGRAS104	TaGRAS105	0.007159	0.048872	0.146482	3.725019
TaGRAS106	TaGRAS107	0.003841	0.054558	0.070411	4.158361
TaGRAS111	TaGRAS112	0.011238	0.148462	0.075699	11.31569
TaGRAS120	TaGRAS121	0.007413	0.104675	0.070822	7.978312
TaGRAS123	TaGRAS124	0.003378	0.052178	0.064735	3.976993
TaGRAS125	TaGRAS127	0.045741	0.089441	0.511402	6.817181
TaGRAS128	TaGRAS129	0.126865	0.206353	0.614798	15.72811
TaGRAS132	TaGRAS133	0.072932	0.117197	0.622302	8.932695
TaGRAS137	TaGRAS142	0.03905	0.058561	0.66682	4.463501
TaGRAS139	TaGRAS140	0.044977	0.090317	0.497991	6.88393
TaGRAS143	TaGRAS144	0.046442	0.076873	0.604133	5.859259
TaGRAS146	TaGRAS147	0.023516	0.05818	0.404194	4.434448
TaGRAS149	TaGRAS150	0.020502	0.059132	0.346721	4.507033
TaGRAS151	TaGRAS152	0.010776	0.046293	0.232773	3.52844
TaGRAS157	TaGRAS158	0.008244	0.047749	0.172648	3.639392
TaGRAS159	TaGRAS160	0.396546	0.473053	0.838269	36.05584
TaGRAS162	TaGRAS163	0.012551	0.046347	0.270811	3.53253
TaGRAS165	TaGRAS166	0.013928	0.073084	0.19058	5.570461
TaGRAS167	TaGRAS169	0.018369	0.054928	0.334424	4.186562
TaGRAS170	TaGRAS171	0.021159	0.108987	0.194148	8.306898
TaGRAS173	TaGRAS174	0.016478	0.082212	0.200438	6.26614

**Figure 4:**
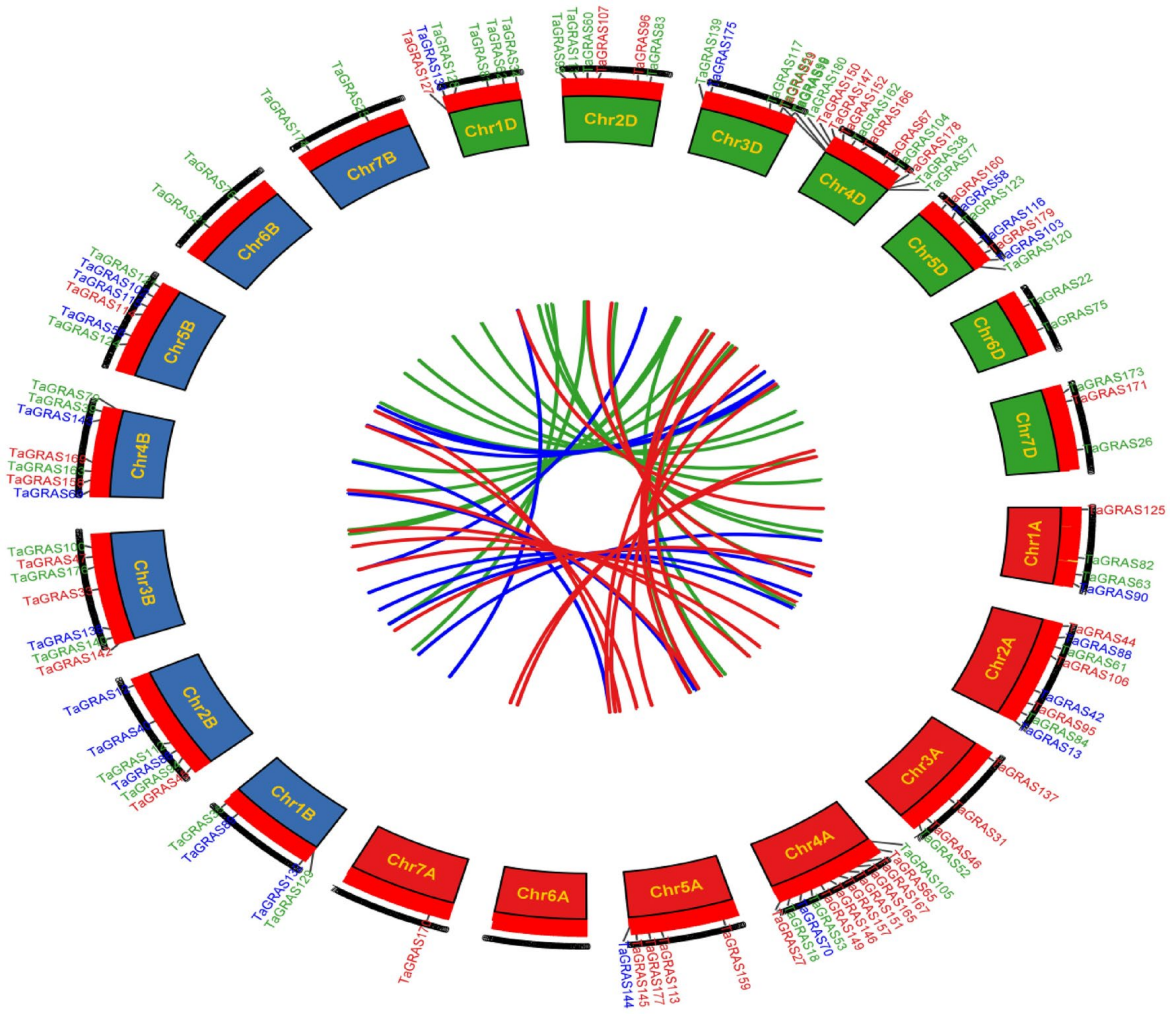
The synteny analysis of TaGRAS family in *T. aestivum*. Different colours represent GRAS subfamilies on A, B and D sub-genome red lines indicate duplicated TaGRAS subfamily gene pairs on A sub-genome, green lines indicated on B sub-genome and blue represented D sub-genome. The chromosome number is indicated at the bottom of each chromosome by using TBtools^41^.

We analyzed nine segmental events on various chromosomes and one tandem duplication event on the same chromosome. The results showed that segmental duplication events were essential for the expansion of *TaGRAS* genes in the wheat genome and that certain *TaGRAS* genes may have been created through gene duplication. The tandem and segmental duplicated genes belong to LISCL, PAT1, DLT, and RAM1. We also investigated how frequently tandem duplications occur. This region contained 60 *TaGRAS* gene pairs, all of which were closely linked. The identities of these, however, were > 80%, indicating that they were incorporated into instances of tandem duplication. Since gene duplication has a significant influence on the creation of novel abilities and gene families, we examined the *TaGRAS* gene duplication events in the wheat genome. The paralogous gene pairs were used to draw the circos (Fig. 4).

### Analysis of promoter sequences in *GRAS* genes in wheat

One hundred and twelve *cis*-elements were identified in the ~ 1.5 kb region upstream of the transcription initiation site of the *TaGRAS g*ene (Supplementary Fig. 5). Several key *cis*-elements including the ABRE (ABA-responsive element), MYB (drought–responsive) and LTRE (low temperatures) were found. It was found that *cis*-element types exist in the genes LISCl, PAT1, DLT, NSP2, and HAM subfamilies. Finally, the *cis*-elements investigation showed that separate subfamily genes may be controlled in diverse ways and that the majority of *TaGRAS* genes can respond to a range of environmental stresses.

### Interaction network of TaGRAS proteins

**Figure 5:**
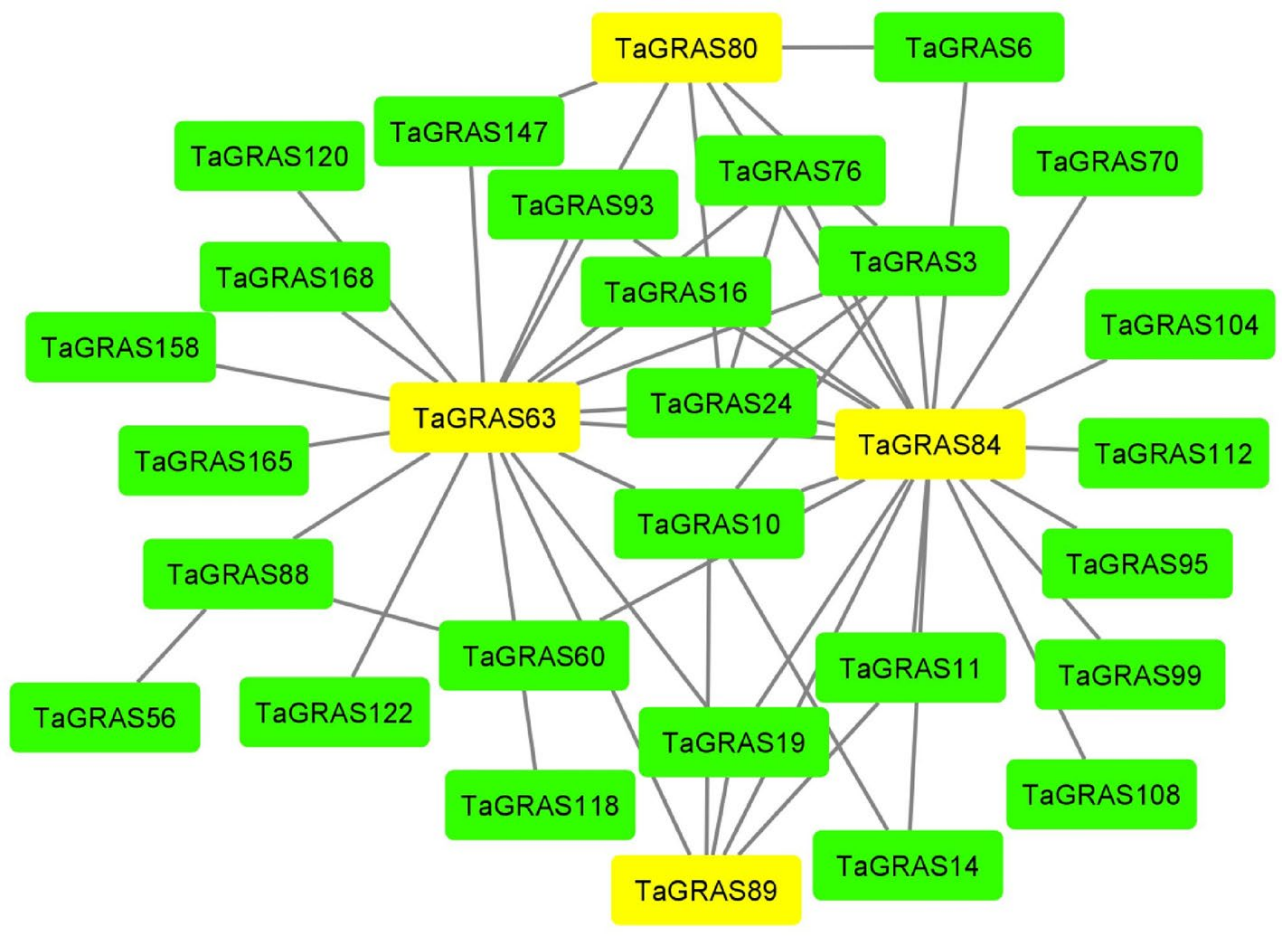
Interaction network among TaGRAS families in wheat. Specific protein interactions between GRAS transcription factors in wheat were determined using String^42^.

In our study, we utilized homology analysis of GRAS proteins in wheat and Arabidopsis via the STRING database to create a protein interaction network for TaGRAS proteins. We identified four key hub genes, TaGRAS63, TaGRAS84, TaGRAS80, and TaGRAS89, based on their high degrees of interactions, specifically TaGRAS18, TaGRAS21, and TaGRAS5, respectively (Fig. 5). These hub genes play significant roles in various biological processes. TaGRAS63 encodes an SCR protein influencing mesophyll and bundle sheath cell development, as well as radial patterning in embryonic roots and shoots. TaGRAS80 is involved in the nodulation signalling pathway, impacting nodulation component production, root development, and interactions with other phytohormones. TaGRAS89, resembling a scarecrow protein with DNA binding transcription factor activity, resides in the nucleus, controlling transcription and gene expression. It interacts with transcriptional activators genes regulating root growth, stamen formation, cell expansion, and flowering time. Lastly, TaGRAS84 interacts with genes such as jasmonate O-methyltransferase (JMT), a key player in plant defence by converting jasmonate into methyl-jasmonate.

### Expression Patterns of *GRAS* genes

To examine the expression profiles of wheat GRAS members in different tissues, we used the TPM values of all *TaGRAS* genes obtained from abiotic stress experiments and different developmental stages. The expression levels of different *GRAS* genes varied greatly within the same tissue (Supplementary Fig. 6). About one-tenth of the 177 *GRAS* genes were not expressed. In this study, five different tissues were obtained at three different developmental stages. Different TaGRAS subfamilies exhibit varying levels of induction in various tissues (Supplementary Fig. 6A). In the Z39 stage, *GRAS* genes such as *TaGRAS27, TaGRAS*28, *TaGRAS*106, *TaGRAS*110, *TaGRAS*111, and *TaGRAS*112 showed more than fivefold up-regulation. However, at the Z75 stage, the expression of genes *TaGRAS27, TaGRAS28, TaGRAS98, TaGRAS100, TaGRAS104, TaGRAS106, TaGRAS107, TaGRAS108, TaGRAS176,* and *TaGRAS179* was up-regulated in leaf tissues. Notably, at the Z39 stage, some *GRAS* genes showed up-regulation. At the Z10 stage, the expression of 16 TaGRAS family genes increased more than threefold root tissues. This suggests that different *TaGRAS* genes may be involved in the development of various tissues at different stages. Many *GRAS* genes, including *TaGRAS98, TaGRAS99, TaGRAS*100, *TaGRAS*106, *TaGRAS107, TaGRAS108, TaGRAS111, TaGRAS112, TaGRAS117, TaGRAS160, TaGRAS164, TaGRAS177*, and *TaGRAS179* showed induction at different stages of the wheat grain aleurone layer. Only a few members of the TaGRAS subfamily showed no induction at any of the several embryonic phases.

We also assessed *in-silico* expression analysis of *GRAS* genes in wheat tissues under abiotic stresses (Supplementary Fig. 6B). Across all examined tissues, *GRAS* genes from the DELLA, PAT1, and LISCL subfamilies exhibited high expression levels (log_2_-based values > 5), suggesting their potential role in tissue development based on their tissue-specific expression patterns. Notably, several genes similar to *AtPAT1* showed strong expressions in leaves, aligning with *AtPAT1's* role as a positive regulator in the phyA signal pathway. Several *TaGRAS* genes showed constitutive expression levels during the majority of the phases of wheat growth. For instance, a > 5-folds increased level was observed for six genes (*TaGRAS27, TaGRAS28*, *TaGRAS98*, *TaGRAS99*, *TaGRAS110*, and *TaGRAS112*) under drought stress, as that of *TaGRAS159, TaGRAS160, TaGRAS171,* and *TaGRAS179* for heat stress. However, *TaGRAS179, TaGRAS160, TaGRAS159,* and *TaGRAS99* showed a considerable level of expression for both stresses. Other *GRAS* genes were active at low expression levels in a variety of tissues and at different developmental stages in wheat. These findings showed that *TaGRAS* genes exhibit a wide range of expression pattern, and that even genes belonging to the same subfamily have distinct expression patterns.

### *TaGRAS* genes are involved in the response to abiotic stresses

**Figure 6:**
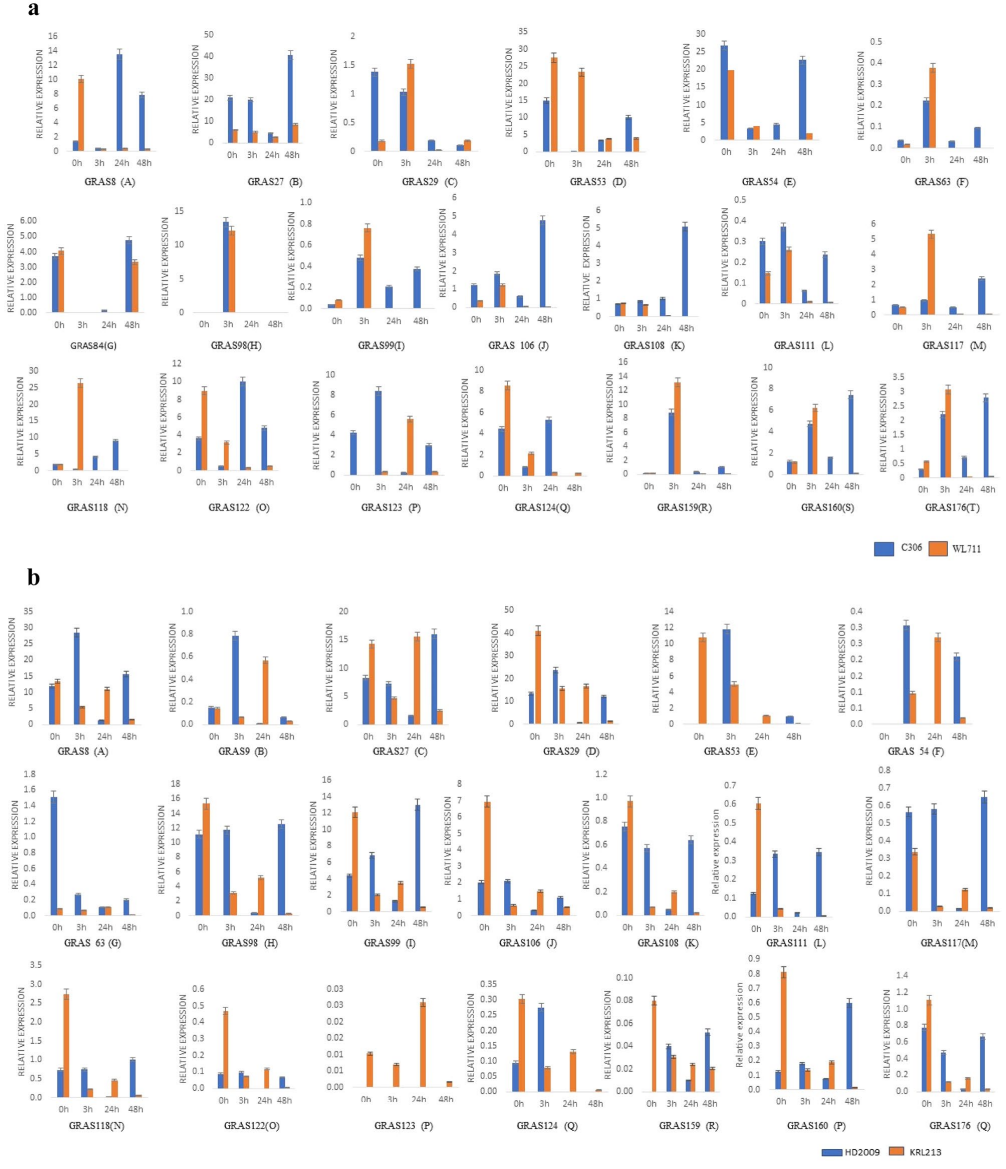

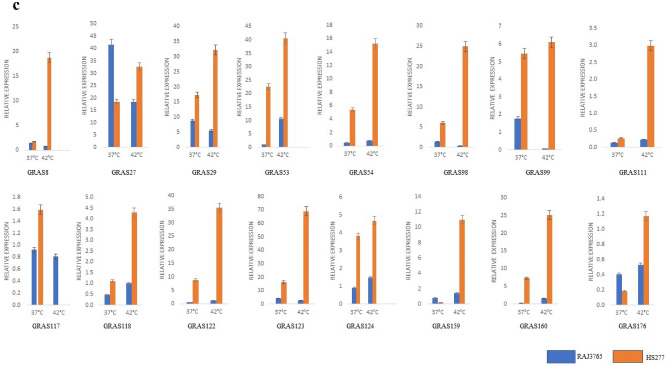
qRT-PCR based differential expression analysis of 20 *TaGRAS* genes under abiotic stress. (**A**) drought stress at 20% (v/v) PEG treatment in C306 and WL711 for 24 h leaf tissues, (**B**) Salt stress treatment at 150 mM NaCl in Kharchia65 and HD2687 at 0 h, 24 h and 48 h leaf tissues, (**C**) Heat stress treatment in Raj3765 and HD2009 at 37 °C and  42 ^0^C.

Overall, many *TaGRAS* genes were significantly induced/repressed by the various forms of stresses as shown in Fig. 6. The expression levels of these genes changed over time or in different tissues depending on the specific treatments. The relative expression levels of six genes (*TaGRAS8, TaGRAS27, TaGRAS53, TaGRAS54, TaGRAS98,* and *TaGRAS122*) significantly showed their expression > tenfold under drought stress. However, the expression of nine genes (*TaGRAS106, TaGRAS108, TaGRAS117, TaGRAS118, TaGRAS123, TaGRAS124, TaGRAS159, TaGRAS160,* and *TaGRAS176*) were not obviously changed, followed by downregulation of *TaGRAS29, TaGRAS63, TaGRAS84, TaGRAS99,* and *TaGRAS111* genes expression (Fig. 6A) in leaf tissues of wheat variety C306. Whereas in root tissues, 19 gene expression levels was more than two-fold. Likewise, the expression level of *TaGRAS8, TaGRAS54* and *TaGRAS159* was ~ 10-folds in leaf tissues followed by expression > threefold in five genes at different time intervals. The expression patterns in root tissue of WL711 showed that eight genes were expressed 15-folds while seven genes showed moderate expression at different time intervals followed by down-regulated of remaining genes (Supplementary Fig. 7).

Under salt stress conditions, the expression level in leaf tissue of HD2009 at 0 h, three genes (*TaGRAS8, TaGRAS98,* and *TaGRAS29*) were expressed more than tenfold and three genes (*TaGRAS27, TaGRAS99,* and *TaGRAS106*) were expressed more than two folds (Fig. 6B). While KRL213 wheat six genes (*TaGRAS8, TaGRAS*27, *TaGRAS29, TaGRAS98, TaGRAS99* and *TaGRAS53*) were expressed more than 10-folds. Further, the expression level in root tissues of HD2009 was shown to be higher only in *TaGRAS27* and *TaGRAS99* and five genes (*TaGRAS8, TaGRAS29, TaGRAS98, TaGRAS106,* and *TaGRAS118*) were moderate. Whereas, expression level in root tissue of KRL213 was found > 20-fold in *TaGRAS8, TaGRAS27,* and *TaGRAS98* (Supplementary Fig. 8). After 3 h of stress, the expression in leaf tissue of HD2009 genotype was more than tenfold in four genes (*TaGRAS8, TaGRAS29, TaGRAS53* and *TaGRAS98*). However, in KRL213, expression of *TaGRAS27* was more than forty-five folds in leaf tissue followed by *TaGRAS29* of ten folds. To check the *GRAS* gene expression in root tissue in HD2009 genotype, it was found that only three genes (*TaGRAS8, TaGRAS27,* and *TaGRAS29*) were expressed more than two folds. Whereas, expression of KRL213 root tissue was ~ 25-folds in *TaGRAS29*. At 24 h expression level was lower in HD2009 than KRL213 in leaf tissues. Whereas, the expression in root tissues two genes expressed more than 30-fold and five genes were expressed more than two fold in the HD2009 genotype. While in the root tissue of KRL213, only one gene (*TaGRAS27*) is expressed more 30-fold and three genes were expressed more than six folds. Expressions at 48 h in leaf tissue of HD2009 were found to be highly expressed in five genes (Fig. 6B) while it was low at KRL213. After that, the expression was calculated in the root tissues of HD2009 and only two genes (*TaGRAS8* and *TaGRAS27*) showed moderate expression (Supplementary Fig. 8) as compared to that of KRL213 where three genes (*TaGRAS8, TaGRAS27* and *TaGRAS29*) were expressed with ~ 15 folds after 48 h of stress. Apart from this, the expression of other genes was very low in both leaf and root tissues.

Similarly, *GRAS* gene expression under heat stress with two contrasting genotypes RAJ3765 (resistant variety) and HS277 (susceptible variety) was studied (Fig. 6C). To check the expression of *GRAS* genes in RAJ3765 under acquired conditions, six genes (*TaGRAS54, TaGRAS98, TaGRAS99, TaGRAS122, TaGRAS124,* and *TaGRAS160*) showed expression up to seven folds more while four genes (*TaGRAS27, TaGRAS29, TaGRAS53,* and *TaGRAS123*) were expressed more than fifteen folds. While in HS277 genotype, it was observed that three genes (*TaGRAS27, TaGRAS29,* and *TaGRAS123*) were found to express more than four folds and four genes (*TaGRAS8, TaGRAS53, TaGRAS98,* and *TaGRAS99*) were expressed more than one-folds. After that, we checked the GRAS gene expression in basal condition and the results showed that three genes (*TaGRAS8, TaGRAS54,* and *TaGRAS159*) were expressed more than ten folds, two genes (*TaGRAS98* and *TaGRAS160*) were expressed more than twenty folds and three genes (*TaGRAS27*, *TaGRAS29,* and *TaGRAS53*) were expressed more than thirty folds. However, in expression level in HS277 under basal conditions, two genes were expressed more than ten folds, one gene expressed up to five folds, and five genes were expressed more than one-folds (Fig. 6C). Understanding the expression patterns of the *TaGRAS* gene about stress provides useful information for their functions in managing abiotic stress.

### MD simulation analysis

**Figure 7:**
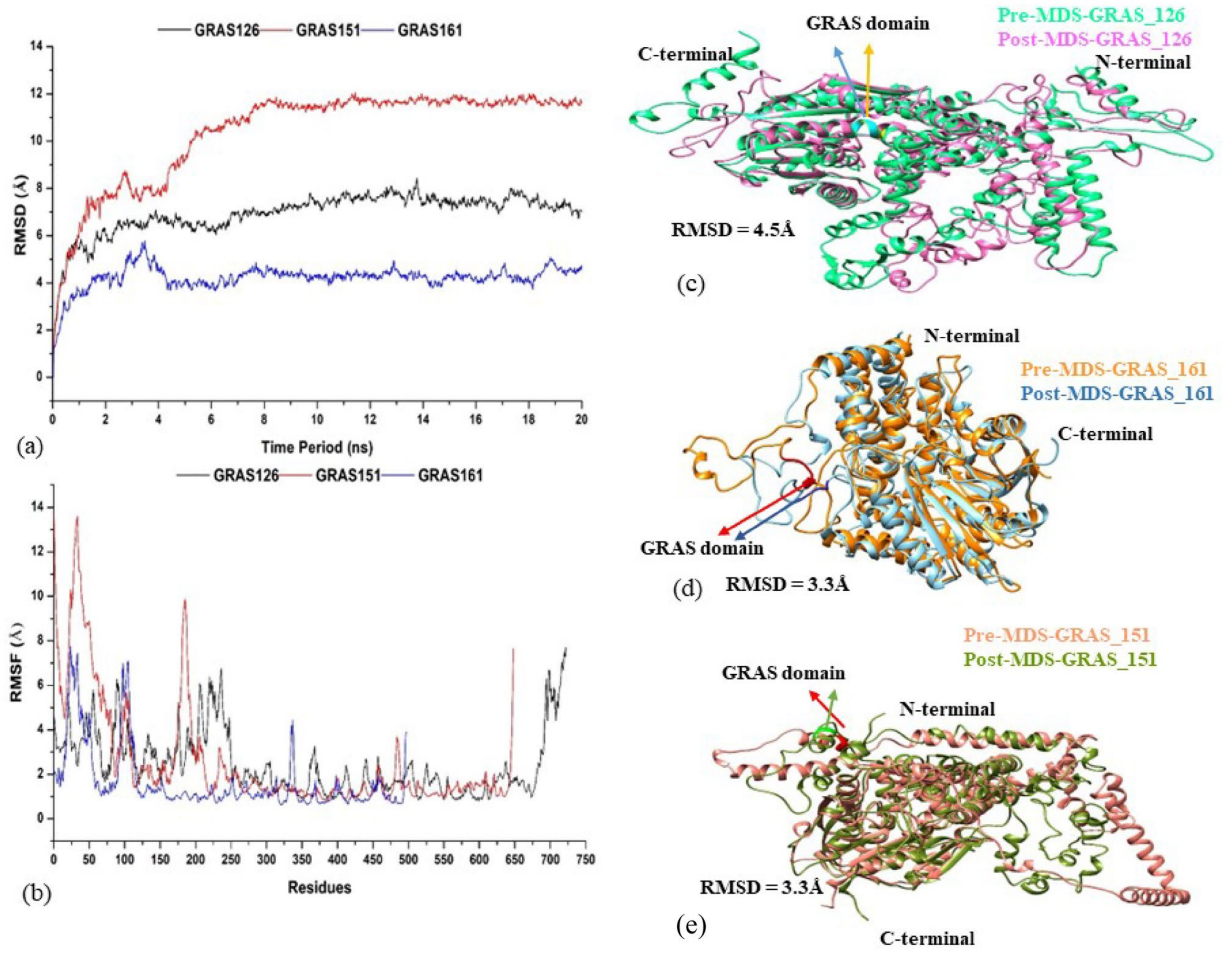
MDS analysis (**a**) Root Mean Square Deviation, (**b**) Root Mean Square Fluctuations, (**c**) Ribbon shape superimposed structure of GARS_126, (**d**) GARS_161, (**e**) GARS_151 are shown, the orange colour structure is the initial phase of the system at 0 ns and the blue colour structure is the final phase of the system after 20 ns of Molecular Dynamics Simulation. GARS domain is shown in red and dark blue for the pre- and post MDS structures generated using the Rosettafold^49^.

The average change in displacement of a particular set of atoms for a given frame relative to a reference frame is calculated using the Root Mean Square Deviation (RMSD). Tracking the RMSD of the protein throughout the simulations can provide information about protein structural conformation. The RMSD number for the backbone atom of the protein concerning its initial positions increased to 7, 10, and 4 Å for GRAS126, GRAS151, and GRAS161, respectively. The large RMSD value for the GRAS126 and GRAS151 indicates these structures were undergoing large structural changes during simulations. Figure 7A showed that the simulation was equilibrated as the RMSD value stabilized ~ 18 ns which provide an appropriate basis for subsequent investigation.

The root mean square fluctuation (RMSF) provides insight into the local changes that occur during the MD simulations in the protein chain. The fluctuations of the major peaks in the RMSF graph were observed majorly at the N-and C-terminal of the proteins. However, no fluctuations were observed in the GRAS motifs in all the proteins. For GRAS151, the backbone residue position between Gly203-Ser206 varies from 6.4 to 7.7 Å, for GRAS126, (Gly396-Ser399) varies from 1.1 to 1.4 Å and for GRAS161, (Gly53-Ser56) it varies from 7 to 10 Å (Fig. 7a,b).

The initial and final frame structure of the proteins was superimposed using DALI server (http://ekhidna2.biocenter.helsinki.fi/dali/) and their deviations are measured in RMSD (Table 3). The secondary structure analysis shows changes in the frequencies, as the helix was found to decrease in the post-MD structure in all GRAS proteins. Furthermore, there was a slight decline in the sheet structure in the GRAS 126, GRAS161 and GRAS151 (Fig. 7c–e). Whereas, change in the secondary structure of GRAS domain was observed only in the GRAS161, (Gly53-Ser56Pre-MD, TTTG; Post-MD, TTTT) (Table 3; Fig. 7d).

**Table 3:** Analysis of secondary structure.

	Systems	Helix (%)	Sheet (%)	Other (%)	Secondary structure	RMSD (Å) b(superimposition)
GRAS_126	Pre-MD	50.6	10.2	39.1	HHHH	4.5
	Post-MD	35.8	10.4	53.8	HHHH	
GRAS_161	Pre-MD	50	13.1	36.9	TTTG	3.3
	Post-MD	38.4	11.4	50.2	TTTT	
GRAS_151	Pre-MD	59.0	9.6	31.4	HHHH	4.4
	Post-MD	44.2	8.6	47.1	HHHH	

## Discussion

Plant genome sequencing has been used to investigate genes involved in various developmental stages and stress tolerance in a wide range of crops. Crops whose genomes have not been sequenced yet are receiving benefits from model plant genomes such as *A. thaliana* and *O. sativa.* Many studies have shown that transcription factors like the *GRAS* gene family play a crucial role in resisting abiotic, biotic, and individual plant growth development^10, 69^. However, the *GRAS* gene family analysis was previously reported in barley (53) cucumber (35), tomato (53), tea (52), soybean (117), *M. sativa* (51), foxtail millet (57), wheat (117), and cotton (15)^70–74^. Nevertheless, few studies have been conducted on wheat too^73, 74^. However, Liu et al.^67^ used transcriptomic data for *in-silico* analysis of 180 GRAS genes using IWGSC RefSeq 1.0 assembly, while Kumar et al.^68^ identified 183 GRAS genes in wheat using the Gramineae database. Both the studies lack in experimental validation of *GRAS* genes and protein–protein interaction. Here, we performed a comprehensive analysis of the 177 GRAS members in wheat using the latest assembly (IWGSC_v2.1), including their phylogenetic relationships, gene structure, conserved motifs, chromosomal positions, molecular dynamics simulation, and expression profile under abiotic stresses.

When compared to previously identified Arabidopsis (33 GRAS) and rice (60 GRAS) genes^11^, a fairly large number of these genes were discovered in wheat (177 GRAS). These findings may justify the fact that wheat has a large genome and has evolved with a higher ploidy level^75^. However, five highly conserved domains were shared with the majority of GRAS genes: LHRI, VHIID, LHRII, PFYRE, and SAW motif (Supplementary Fig. 4). These motifs have previously been shown to affect protein–protein and protein-DNA interactions^13^. The structural study revealed that the majority of the GRAS genes (72%) in the subfamilies (SHR, HAM1, LAS, and SCL4/7) were free from introns in line with studies conducted in *Arabiddopsis*, rice, *Brassicsa*, and *Medicago*^5, 76^. This exon–intron structure found in *TaGRAS* genes was comparable to those seen in other species^77, 78^. Intron genes are abundant in several large gene subfamilies (LISCL, PAT1, and SCR)^79^. Nonetheless, distinct exon–intron structures have formed in several GRAS genes, indicating that they have likely acquired new specialized roles to adapt to their environment. According to previous studies, the plant *GRAS* gene family evolved from a prokaryotic genome by horizontal gene transfer, followed by duplication events^80^. Many *TaGRAS* genes have major outliers with more than 5 introns, demonstrating the *TaGRAS* gene high degree of divergence. These increases and losses of GRAS might be consequences of chromosomal rearrangement and fusion, resulting in functional diversity across gene families. Introns can prolong genes and increase the frequency of gene combinations. However, intron-less genes lack these advantages in gene combination yet respond quickly to stress^81, 82^. As a result, some *TaGRAS* genes may respond rapidly to environmental signals. The findings in *S. bicolor*, *B. napus,* and *M. truncatula*^81, 83^ and the classifications of *TaGRAS* were comparable, but they were distinct from the reports of eight subgroups in tomato and Chinese cabbage^84^. LISCL had the most GRAS members, which was consistent with previous findings in Arabidopsis, rice, Populus^78^, sorghum^28^, and *H. vulgare*^71^, implying that the gene family may have strong differentiation abilities in the long-term evolution processes. TaGRAS classifications were further supported by their conserved motifs with close TaGRAS from the same subfamilies having similar motif compositions. Because GRAS transcription factors with diverse activities have been widely documented, it is worth noting that various occur in certain subgroups, hinting that they may have unique roles^10, 69^. For example, the DELLA domain, which is located at the N-terminus of members of the DELLA subfamily, may interact with the GA receptors to recognise GA signals^85^. Synteny analysis was also analyzed to assess the relationship between *TaGRAS* genes and their counterparts from *A. thaliana*, *A. tauschii*, *T. diccoides,* and *O. sativa*, representative of Brassicaceae and Poaceae families. The number of orthologous genes discovered between wheat and rice was the highest, suggesting a tight evolutionary relationship, following with *T. diccoides*, *A. tauschii*, and Arabidopsis. These genes might have originated from a common ancestor^28^. Furthermore, complex interactions such as single to many *TaGRAS* genes were discovered, showing that these *T. aestivum* members may play a significant role in *TaGRAS* gene evolution. Furthermore, we discovered that certain *GRAS* genes were only conserved in a few plants; a similar finding was also discovered in sorghum^28^. These findings may be linked to the evolutionary relationships of wheat and also other plant species. Large-scale duplication events, which occur before the divergence of some plant species, also played a crucial role in the expansion of the *GRAS* gene family.

The phylogenetic tree and *cis*-element analyses also provided additional evidence in favour of the possible functions of the *TaGRAS* genes in stress tolerance. Functional characterizations of *GRAS* genes have shown the conserved roles of probable orthologs in each subgroup^84^. Previous reports demonstrated that members of this group, PAT1 (*AthGRAS6*)^86^ and SCL13 (*AthGRAS13*), were involved in the phytochrome A and B signal transduction, respectively^86, 87^. Given that the SHR protein (TaGRAS83-TaGRAS94) from this group may alter root radial patterning and growth, the protein activities of this group may be connected to root development based on alignment with Arabidopsis^14, 88^. The Arabidopsis root/ meristem's cells must be maintained to allow for the plant tissue's indefinite expansion, and SCR, which is downstream of SHR, is necessary for this^14^.

SCL14, an Arabidopsis LISCL subgroup member, interacted with TGA-TFs, required for the activation of the stress-inducible promoters^4^. As a result, several genes in the LISCL subgroup, such as *TaGRAS120, TaGRAS121, TaGRAS124, TaGRAS131, TaGRAS151, TaGRAS161* and *TaGRAS176* were strongly stimulated by multiple stresses and therefore might be implicated in the regulation of stress response pathways (Fig. 5). Furthermore, various stress- and hormone-associated *cis*-elements including MBS, LTR, ABR, and TCA elements have been discovered in the promoter of most *TaGRAS* genes. The findings agreed with earlier studies of GRAS in *B. juncea*^89^, *C. sativus*^29^, and *G. max*^90^.

miRNAs are short noncoding RNAs that regulate cell function at the post-transcriptional and translocation mechanisms. This leads to the degradation of the gene target^91^. In this study, 14 miRNAs involving sequences of *GRAS* genes were discovered (Table 1). Among the predicted miRNAs, miR171 controls the expression of the HAM subfamily by directly targeting the HAMs mRNA^92, 93^. Overexpression of miR171 induced floral transition and spikelet morphological defects in various species, including rice and barley, comparable to Arabidopsis^94, 95^. This provides indirect evidence for the conservation of HAM and its upstream regulator miR171in crops such as barley. The likely targets of wheat miR171 were identified as *TaGRAS82, TaGRAS81, TaGRAS80, TaGRAS79, TaGRAS77, TaGRAS78, TaGRAS74, TaGRAS76, TaGRAS75,* and *TaGRAS73*.

The wheat *TaGRA*S genes are predicted to be involved in stress tolerance or growth and development through a complex protein-interaction network by the STRING database^68^. The conserved GRAS domain is essential for the dimerization of GRAS members and other proteins. In Arabidopsis, the homologous gene GAI from wheat *TaGRAS27, TaGRAS28, TaGRAS29*, and *TaGRAS3*0 have been reported to be involved in reducing ROS accumulation in response to stress, and GAI may interact with several GRAS proteins, including PAT1 (*TaGRAS106, TaGRAS108*, and *TaGRAS176*), SCL3 (*TaGRAS6, TaGRAS10*, and *TaGRAS14*), and RGA1 (*TaGRAS31, TaGRAS32, TaGRAS33* and *TaGRAS34*), indicating that their counterparts in wheat might tend to form similar proteins complexes. The role of GRAS transcription factor in modifying plant responses in a variety of adverse environmental conditions has been extensively documented^67, 77^, indicating that GRAS is an interesting candidate for enhancing plant stress tolerance through molecular breeding. *OsGRAS23* over-expression, for example, increased rice drought and oxidative stress tolerance through modulating stress-responsive genes^96^, while *PeSCL7* over-expression in Arabidopsis demonstrated drought and salt tolerance^27^. Tomatoes silenced by *SiGRAS6 s*howed lower tolerance to drought stress^97^. The relevance of wheat *GRAS* genes in modulating stress response is currently unknown. In this study, transcriptome data and qRT-PCR results demonstrated that the majority of the identified *TaGRAS* genes exhibited significant differential expression under a variety of abiotic stressors, indicating that wheat *TaGRAS* genes can also play critical and diverse roles in response to environmental pollutants. For example, the expression of many *TaGRAS* genes, including *TaGRAS106*, *TaGRAS108*, and *TaGRAS176*, were significantly elevated. The findings show that these *TaGRAS* genes might be important players in the stress response. Previously, it was discovered that *B. rapa* GRAS TF *BrGRAS* is implicated in drought stress tolerance via an ABA-dependent signaling cascade^18^. Furthermore, at least two abiotic stresses simultaneously could be upregulated by the transcription of multiple *TaGRAS* genes indicating that they may have conserved functions in response to these stresses but additional experimental verification is needed.

Protein-DNA interactions are critical in translating genetic information to biological function. Because protein recognition of specific DNA sequences is very complicated, experimental attempts to predict how certain proteins interact with DNA are challenging. As a result, the use of time- and cost-effective computational approaches such as MD simulations and docking studies are essential at this point to accelerate knowledge recovery and limit the search process for experimental protocols. The results of three predicted GRAS proteins using MD simulations revealed complete dynamic and structural information about GRAS domain-DNA interactions. However, there is no structural base information reported to date for GRAS variants and their mechanism of interaction with DNA. However, except of one GRAS protein** (**GRAS161), there was no change in the protein secondary structure and the GRAS motif did not vary significantly across all three GRAS protein studied. Furthermore, the current results of stable structures will pave the way for researchers to investigate the interaction between the GRAS domain and DNA and discover critical residues involved in maintaining the interaction. Our findings showed the importance of selecting a sequence to develop newer transgenic plants that would be increasingly tolerant to stress conditions.

## Conclusion

177 wheat GRAS genes were identified and phylogenetically divided into 14 subfamilies. We discovered that tandem and segmental duplication played a role in the growth of the TaGRAS family. We also discovered that miRNA171, which had previously been reported to have a regulatory function in GRAS member expression, has target genes in *T. aestivum*, all belonging to the HAM subgroup. Finally, qRT-PCR expression results demonstrated that GRAS member’s interacted during response against drought, salt, and heat stress. *TaGRAS*27 might be useful for abiotic stress tolerance for breeding in wheat. This interaction study between the GRAS domain and DNA will identify the key residues important for stabilising the interaction. These findings were critical in understanding the molecular and evolutionary processes of GRAS-mediated plant growth development in wheat.

### Supplementary Information

Supplementary Figures.

Supplementary Table S1.

Supplementary Table S2.

Supplementary Table S3.

Supplementary Table S4.

Supplementary Table S5.

Supplementary Table S6.

Supplementary Table S7.

## Data Availability

All data generated or analysed during this study are included in this published article [supplementary files].
