# Supplementary material for: Genome-wide identification and expression analysis of the GRAS gene family under abiotic stresses in wheat (Triticum aestivum L.)
Source: Sci Rep. 2023 Oct 31;13:18705. doi: 10.1038/s41598-023-45051-0 (PMC10618205; doi:10.1038/s41598-023-45051-0)
Supplement: Supplementary file 1 — Supplementary Figures. [file 41598_2023_45051_MOESM1_ESM.docx]

**
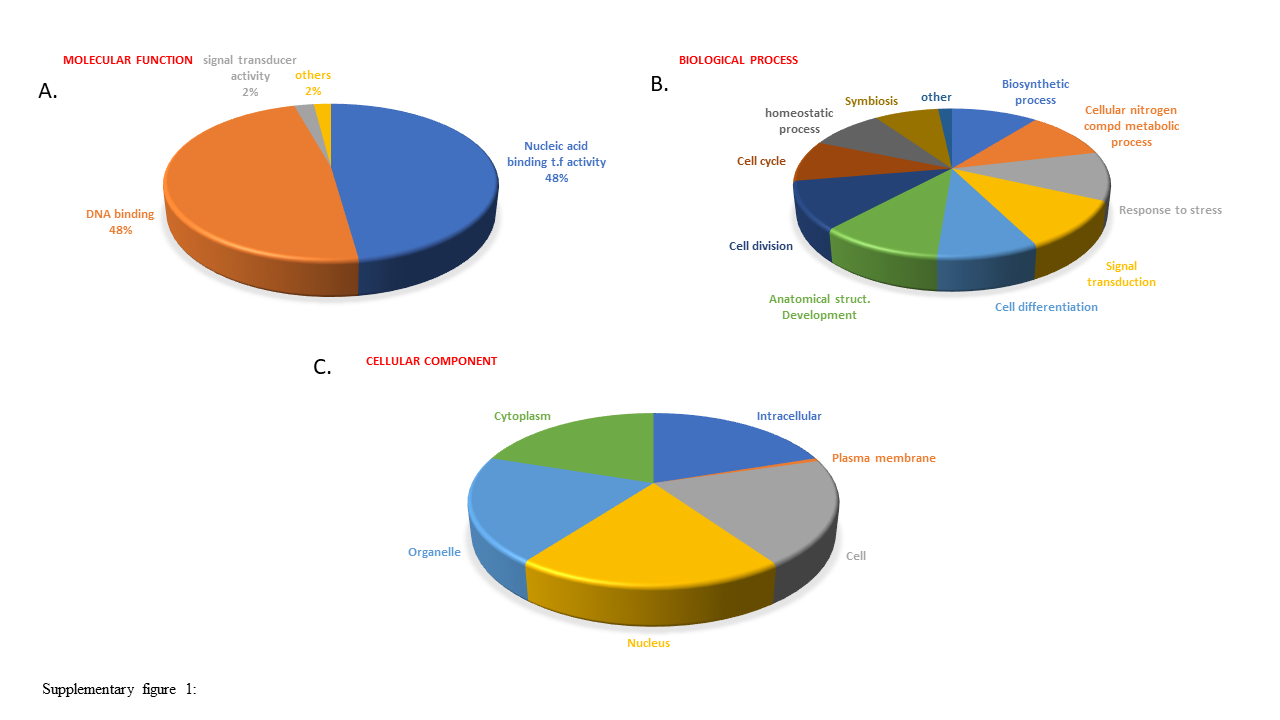
**

**Supplementary Figure** 1: GO enrichment of TaGRAS transcription factors in wheat. The annotation results are divided into three ontology categories using Blast2GO mapping (https://www.blast2go.com/) software. A) represents molecular functions, B) biological processes and C) cellular components .


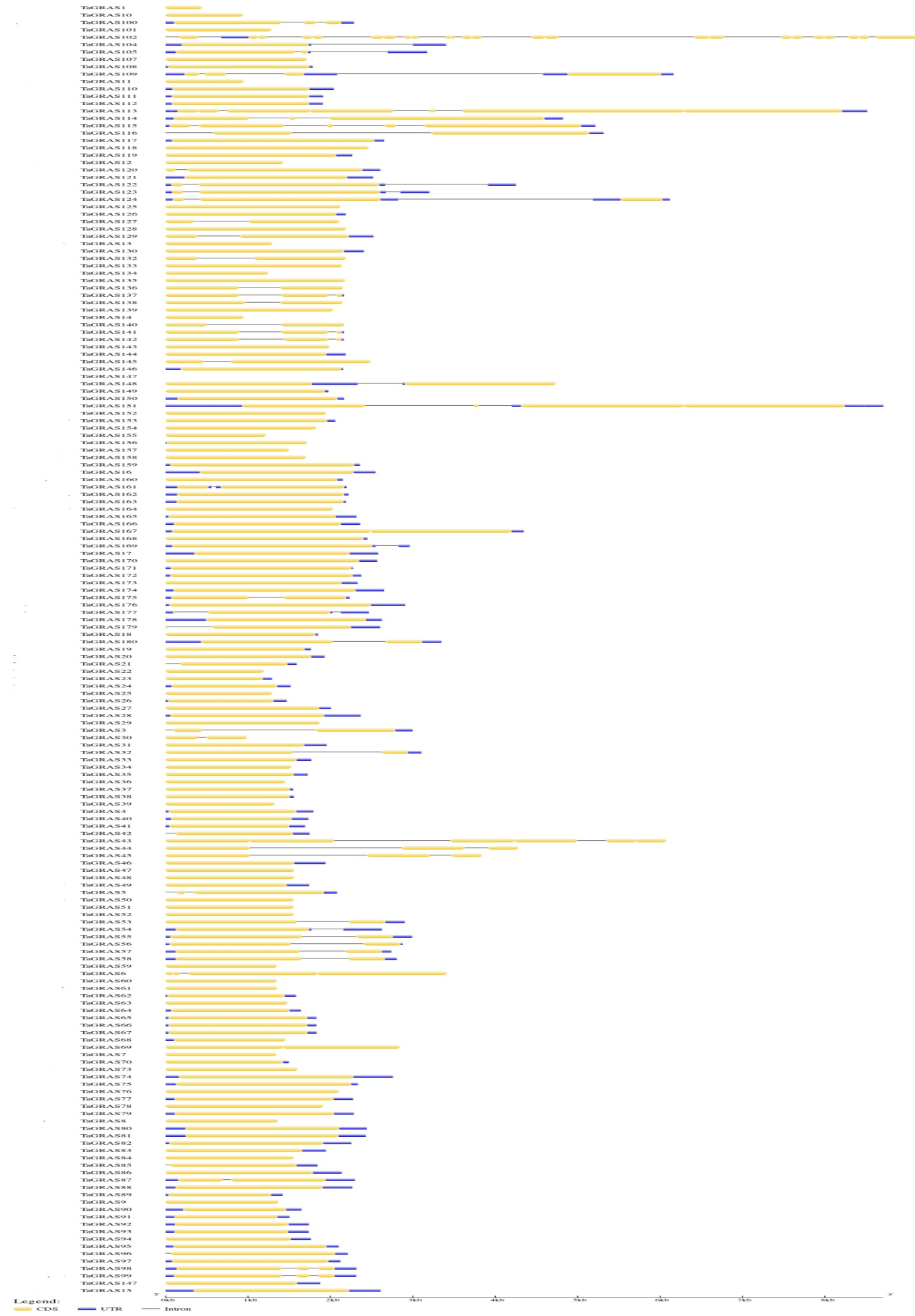


**Supplementary Figure 2:** Gene structure of *TaGRAS* genes family in wheat using Gene Structure Display Server (GSDS 2.0; http://gsds.gao-lab.org/) ^37^, UTR is represented by blue colour, CDS region is marked in yellow and intron in black line.


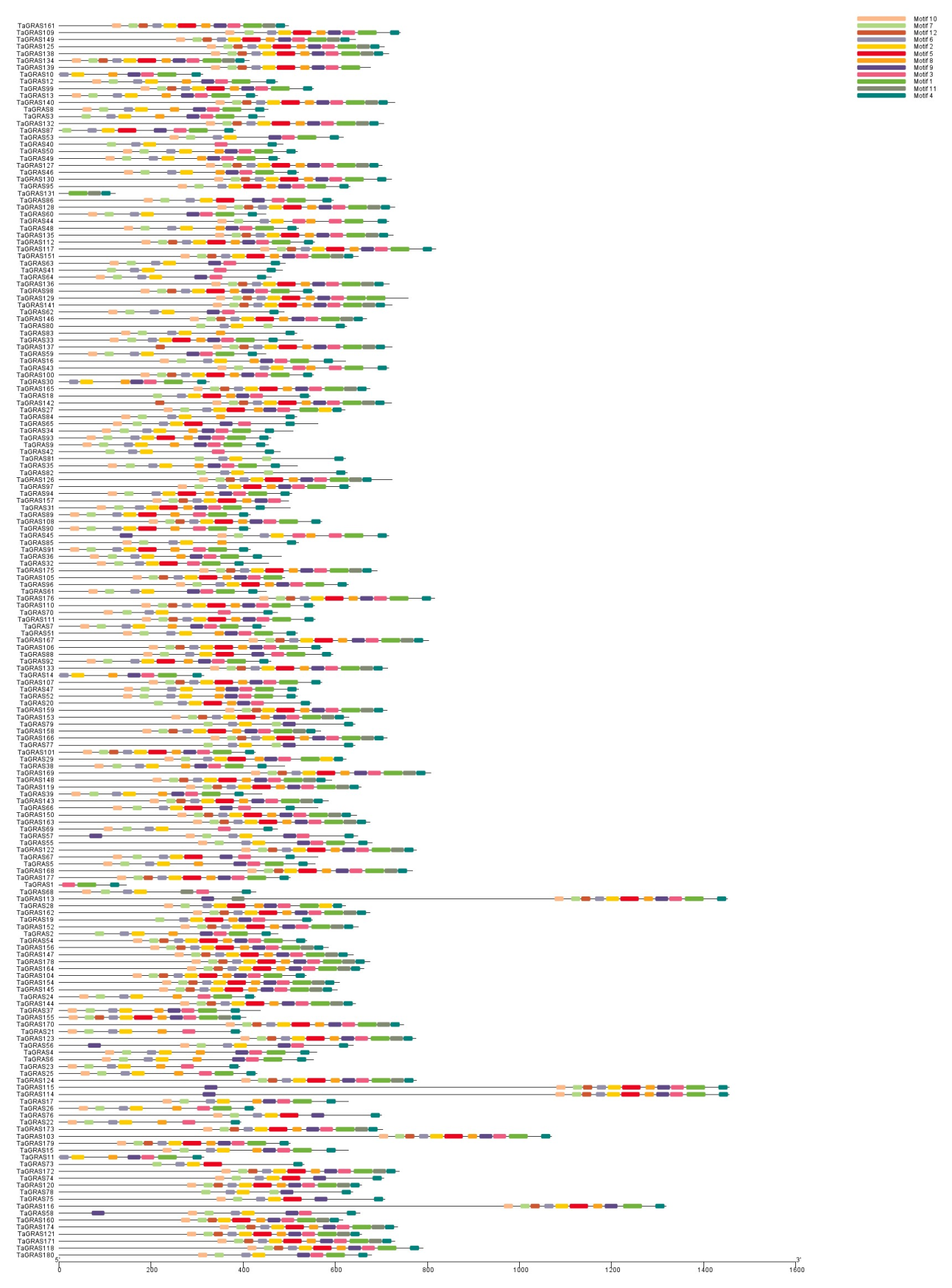


**Supplementary Figure 3**: GRAS structure contains variable region in N-terminus and conserved region in C-terminus, numbers indicate the motif relevant to the domain. The motif distribution of TaGRAS was generated by MEME^36^. Each categorized motif logo was displayed in different box colours.


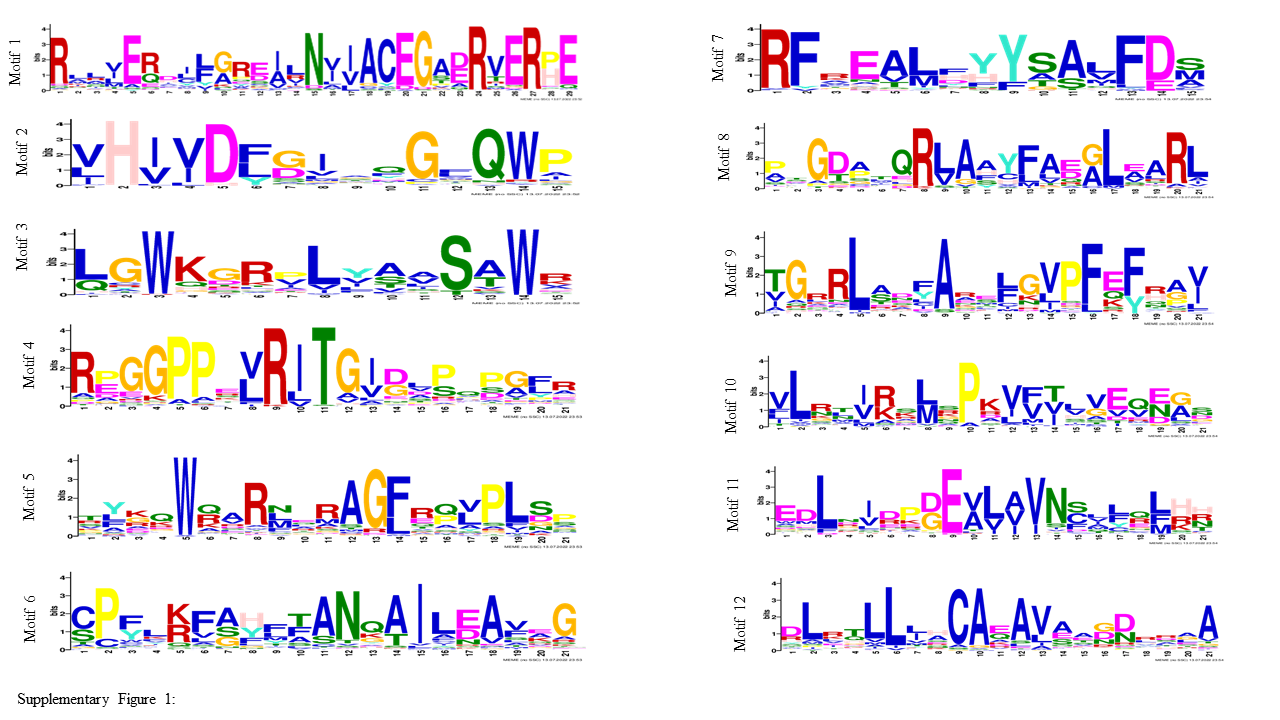


**Supplementary Figure 4:** Amino acid sequences of motifs in logos format


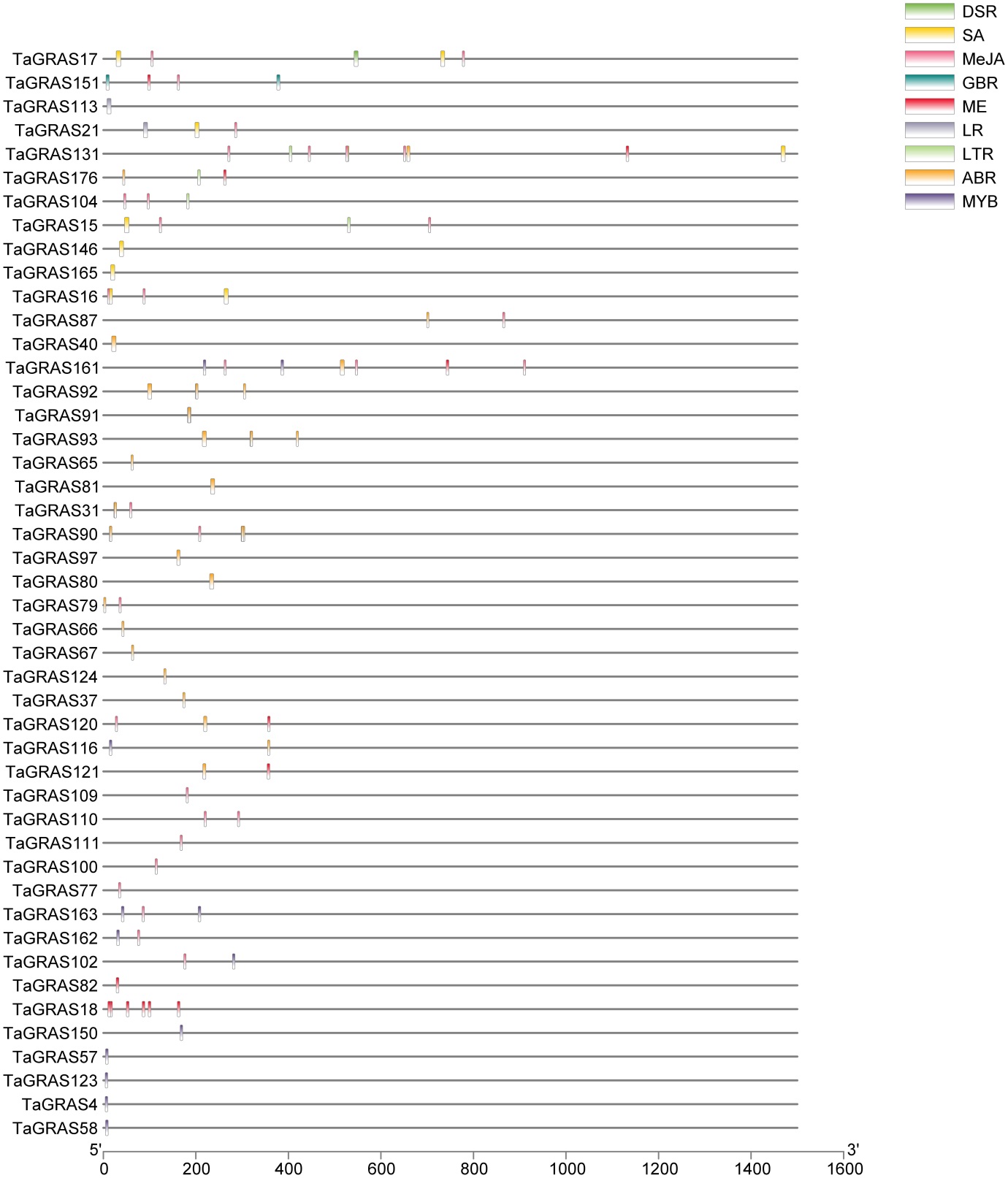


**Supplementary Figure 5:** Predicted cis-elements in TaGRAS promoters. Promoter sequences (− 1500 bp) of 112 TaGRAS genes were analyzed by PlantCARE (http://bioinformatics.psb.ugent.be/webtools/plantcare/html/). The upstream length to the translation starting site can be inferred according to the scale at the bottom. The green, yellow, pink, red, orange and violet-coloured boxes stand for DSR, SA, MeJA, Me, ABRE and MYB cis-elements, respectively.

A)


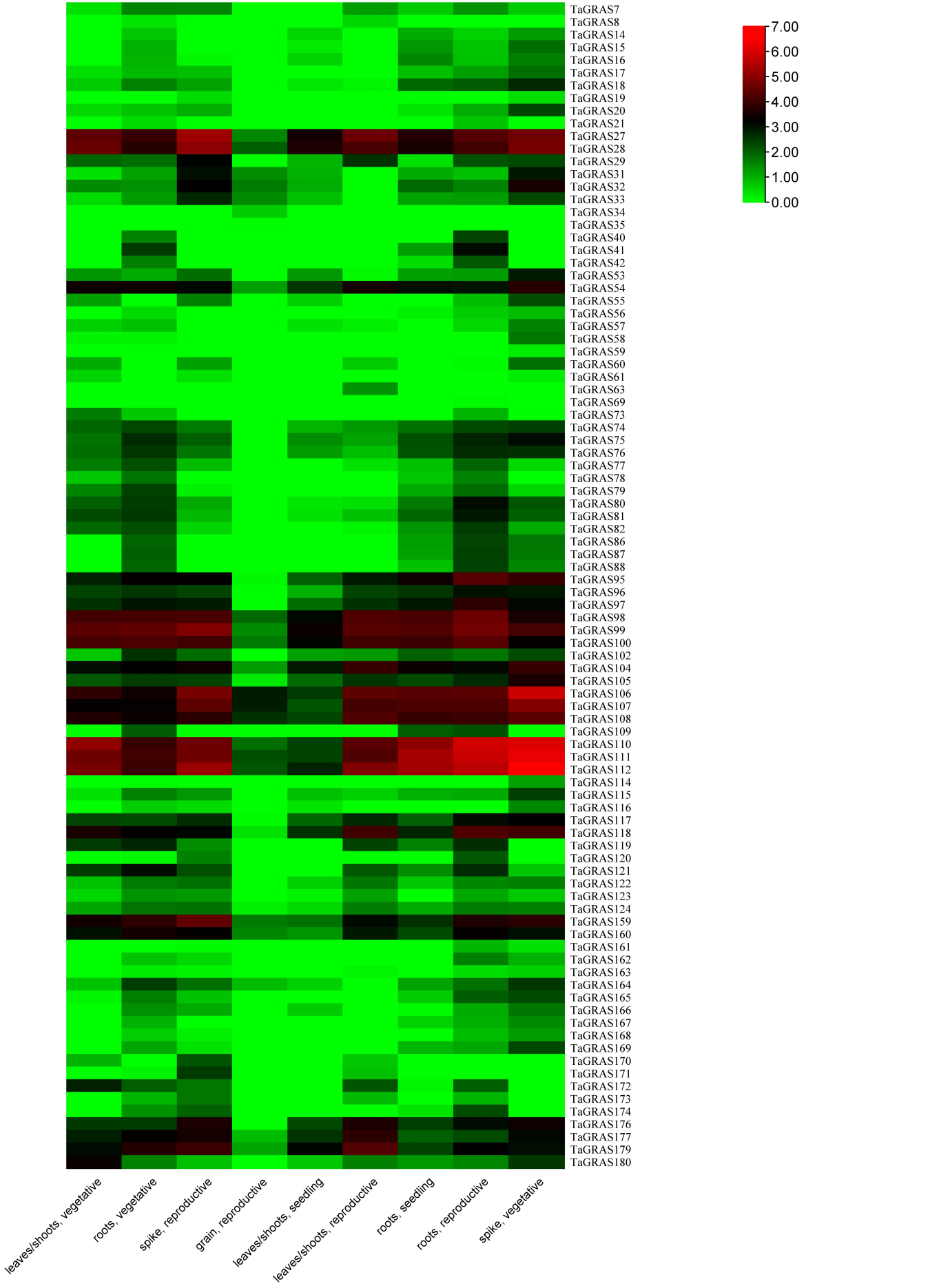


B)


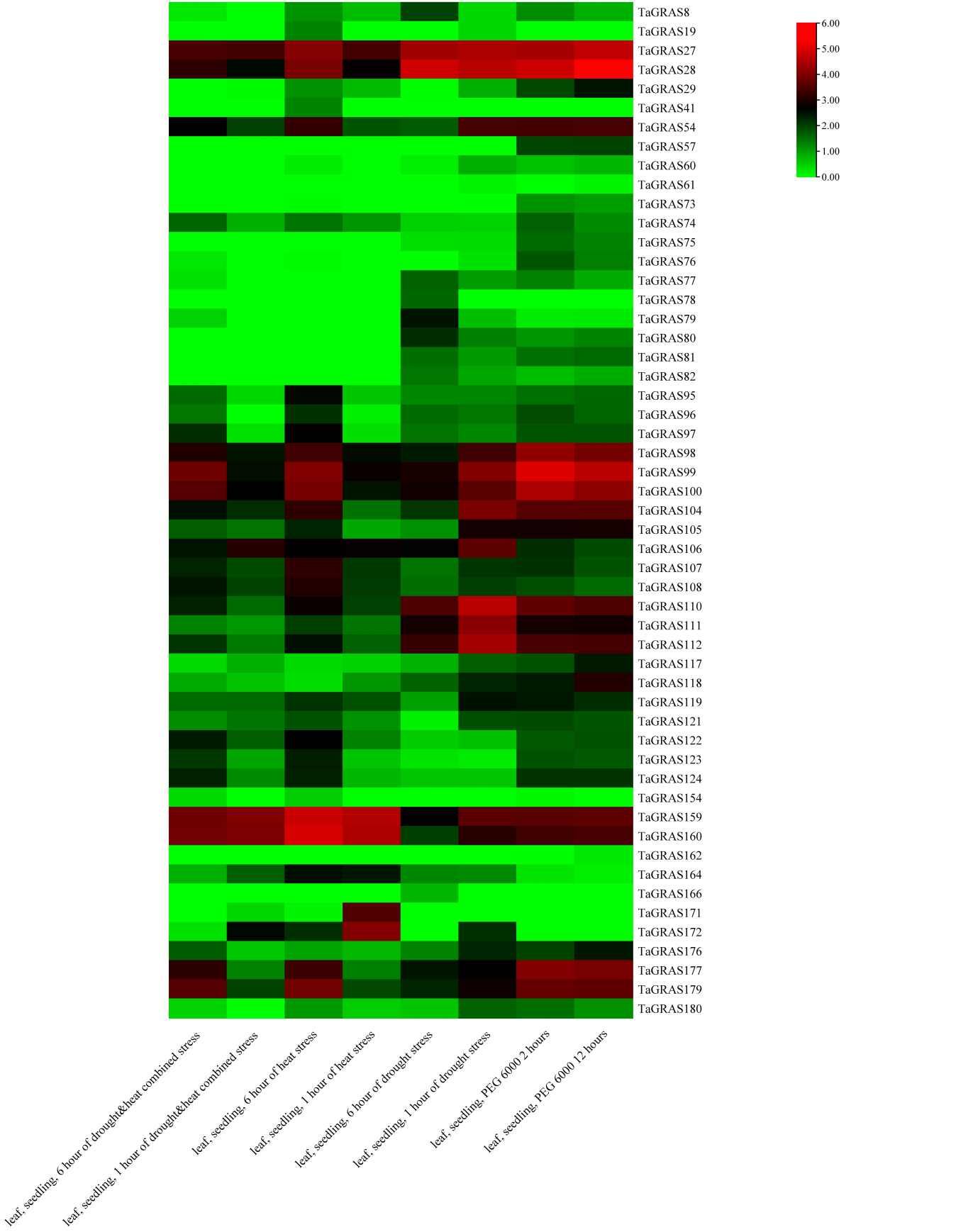


**Supplementary Figure 6**: Heatmap of *TaGRAS* genes (A) tissue specific expression in leaf, root, shoot and spike at seedling, vegetative and reproductive stages (B) abiotic stress specific expression under drought, heat and osmotic stress at seedling stage. The colour scale represents Log_2_ expression values generated using Clustvis (https://biit.cs.ut.ee/clustvis/) ^48^.


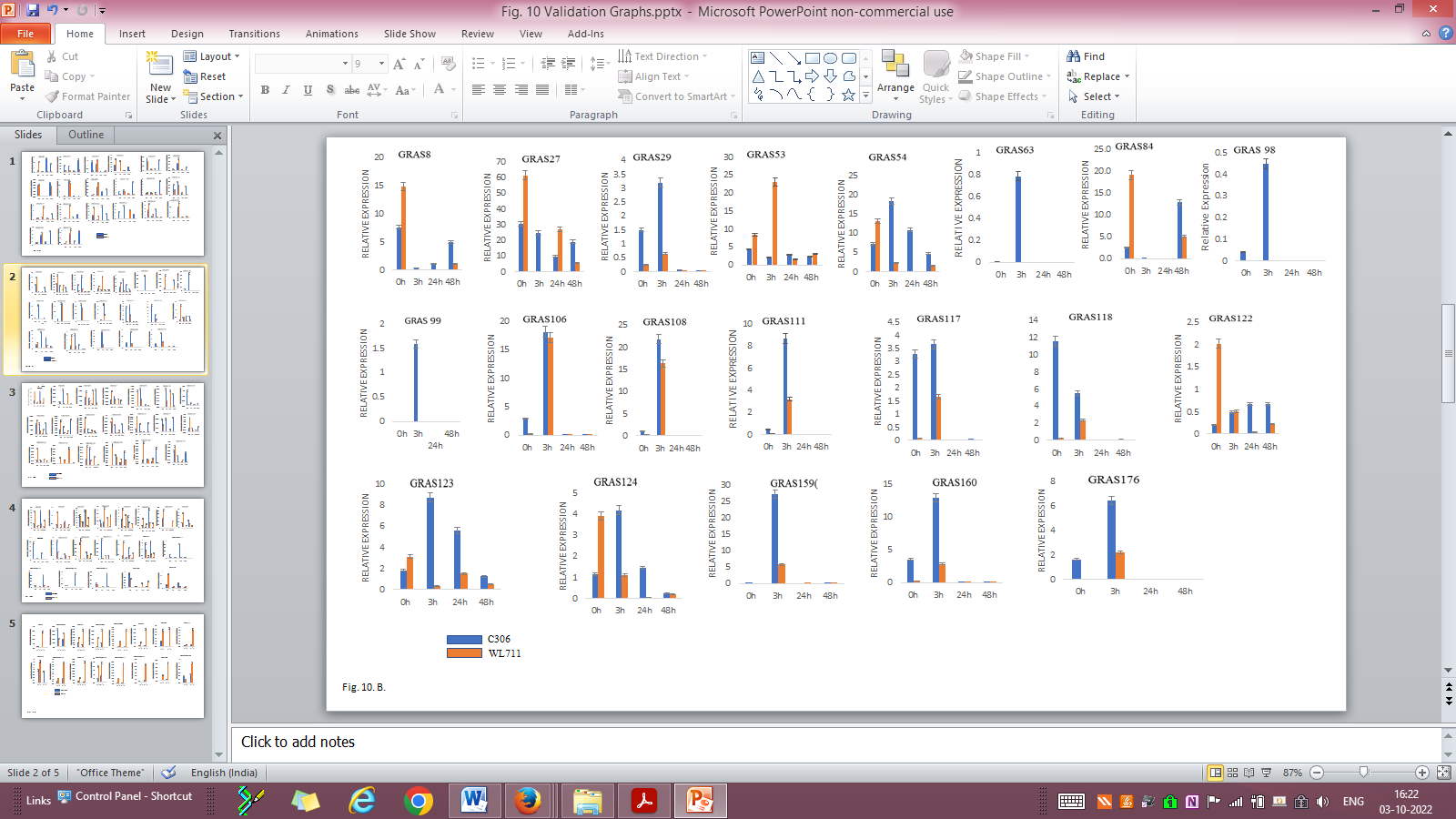


**Supplementary Figure 7:** qRT-PCR based differential expression analysis of 20 *TaGRAS* genes under abiotic stress. A) drought stress at 20% (v/v) PEG treatment in C306 and WL711 for 24 h root tissues.


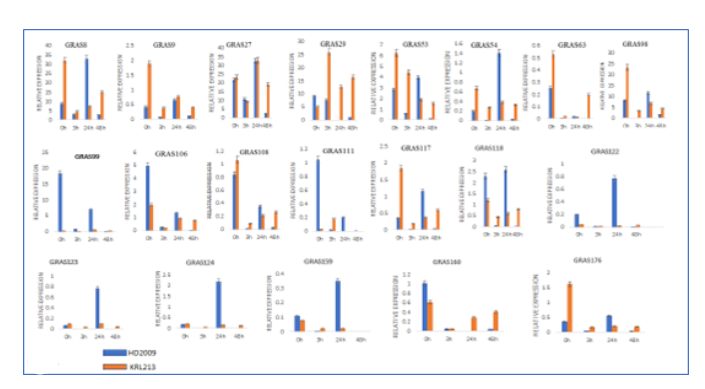


**Supplementary Figure 8:** qRT-PCR based differential expression analysis of 20 *TaGRAS* genes under abiotic stress. Salt stress treatment at 150 mM NaCl in Kharchia65 and HD2687 at 0h, 24h and 48h in root tissues.
